# Caffeine Release from Magneto-Responsive Hydrogels Controlled by External Magnetic Field and Calcium Ions and Its Effect on the Viability of Neuronal Cells

**DOI:** 10.3390/polym15071757

**Published:** 2023-03-31

**Authors:** Emilli C. G. Frachini, Jéssica S. G. Selva, Paula C. Falcoswki, Jean B. Silva, Daniel R. Cornejo, Mauro Bertotti, Henning Ulrich, Denise F. S. Petri

**Affiliations:** 1 Departament of Fundamental Chemistry, Institute of Chemistry, University of São Paulo, São Paulo 05508-000, Brazil; 2 Departament of Biochemistry, Institute of Chemistry, University of São Paulo, São Paulo 05508-000, Brazil; 3 Institute of Physics, University of São Paulo, São Paulo 05508-090, Brazil

**Keywords:** alginate, gelatin, magnetic hydrogel, drug delivery, caffeine, Ca^2+^, SH-SY5Y cells

## Abstract

Caffeine (CAF) is a psychostimulant present in many beverages and with rapid bioabsorption. For this reason, matrices that effectuate the sustained release of a low amount of CAF would help reduce the intake frequency and side effects caused by high doses of this stimulant. Thus, in this study, CAF was loaded into magnetic gelatin/alginate (Gel/Alg/MNP) hydrogels at 18.5 mg/g_hydrogel_. The in vitro release of CAF was evaluated in the absence and presence of an external magnetic field (EMF) and Ca^2+^. In all cases, the presence of Ca^2+^ (0.002 M) retarded the release of CAF due to favorable interactions between them. Remarkably, the release of CAF from Gel/Alg/MNP in PBS/CaCl_2_ (0.002 M) at 37 °C under an EMF was more sustained due to synergic effects. In PBS/CaCl_2_ (0.002 M) and at 37 °C, the amounts of CAF released after 45 min from Gel/Alg and Gel/Alg/MNP/EMF were 8.3 ± 0.2 mg/g_hydrogel_ and 6.1 ± 0.8 mg/g_hydrogel_, respectively. The concentration of CAF released from Gel/Alg and Gel/Alg/MNP hydrogels amounted to ~0.35 mM, thereby promoting an increase in cell viability for 48 h. Gel/Alg and Gel/Alg/MNP hydrogels can be applied as reservoirs to release CAF at suitable concentrations, thus forestalling possible side effects and improving the viability of SH-SY5Y cells.

## 1. Introduction

Caffeine (1,3,7-trimethylxanthine, herein termed CAF) is a natural alkaloid that reversibly blocks the action of adenosine receptors and promotes dopamine release in the central nervous system (CNS) [1]. The U. S. Food and Drug Administration (FDA) has indicated that adult consumers of CAF should limit their intake to 400 mg per day to avoid negative effects [2]. Clinical studies have demonstrated that (i) the gastrointestinal absorption of CAF from an aqueous solution is complete and very fast (30–45 min); (ii) the plasma concentration peaks in ~30 min, resulting in 10 μg/mL [3]; and (iii) the half-lives of CAF and paraxanthine are 4.3 h and 7.8 h, respectively [4]. The administration of low levels of CAF is advised to avoid adverse effects such as the “crash effect”, which occurs after ingesting moderate or high doses of CAF and may include irritation and lack of performance [5,6]. According to Wilhelmus and coworkers, the administration of up to 60 mg of CAF in healthy male and female adults at intervals of up to 45 min may improve performance related to attention, alertness, and mood [7]. However, CAF administration also affects the viability of neuronal cells. For instance, 2.5, 5, 10, and 20 mM of CAF decreased the viability of SH-SY5Y neuroblastoma cells to 72.8%, 65.7%, 46.7%, and 35.7%, respectively [8]. The viability of SK-N-MC neuroblastomas and PC-12 cells, corresponding to a neuronal-like phenotype, was reduced to 50% after 24 h contact with 10 mM of CAF [9] and 5.1 mM of CAF [10], respectively. On the other hand, the incubation of SH-SY5Y [11], HT-22, and BV-2 [12] cells with low doses of CAF (10–500 µM) reduced the formation of reactive oxygen species (ROS), thereby engendering anti-inflammatory and cytoprotective effects. Therefore, the development of biocompatible reservoirs that effectuate the release of CAF at low doses is of interest not only for therapeutic purposes but also for improving the viability of neuronal cells. 

Hydrogels are 3D structures that originate from physical or chemical crosslinked macromolecules and absorb large amounts of water [13]. The combination of hydrogels and magnetic particles yields magneto-responsive hydrogels, which can be manipulated under an external magnetic field [14]. Polysaccharides and proteins are excellent materials with which to build magneto-responsive hydrogels for drug delivery systems due to their non-toxicity, biocompatibility, and relevant role in biological processes [15]. Hydrogels composed of proteins such as fibronectin, gelatin, collagen, laminin, elastin, and silk have been widely applied in the biomedical field [16]. Collagen is the major component of connective tissues such as skin, tendon, and bone. The hydrolysis of the tertiary structure of collagen produces gelatin. This protein is composed of 20 amino acids and its sequence differs from one source to another [17]. Gelatin (Gel) is one of the most common proteins for 3D cell culture due to its biocompatibility and biodegradability. However, due to its poor mechanical properties and fast enzymatic degradation, Gel has been combined with polysaccharides [18] and magnetic nanoparticles (MNP) [19] to form devices with improved properties. Alginate (Alg) is a polysaccharide composed of 1,4-linked β-D-mannuronic acid (M) and α-L-guluronic acid (G) monomers [20]. In the presence of divalent cations, the G-G sequences form stable complexes due to so-called “egg box junctions” [21]. Exposure to magnets constitutes a non-invasive, low-cost stimulus that can modulate the rate at which a drug is released from magnetic matrices [14,22,23,24,25,26]. Usually, the rapid release of CAF from these matrices is due to CAF’s weak interaction with the polymer matrix and its high solubility in water [27,28]. Therefore, chemical and/or magnetic stimuli might improve the sustained release of CAF. Alg hydrogels embedded with MNP improved the sustained release of levodopa [29] and dopamine [30] when exposed to an external magnetic field (EMF). Alg has been applied for the in vitro release of CAF in combination with other compounds. Some examples include Alg/CaCO_3_ hydrogel colloidosomes [31], chitosan-coated alginate microhydrogels [32], and Alg beads combined with pectin, carrageenan, chitosan, and psyllium husk [27]. 

The effects of CAF on humans or cells in culture might be positive or negative depending on the concentration. However, there is a lack of relevant information in the literature regarding stimuli-responsive hydrogels capable of releasing CAF at suitable concentrations for adequate therapeutic administration and improved neuronal cell viability. In this work, magnetic hydrogels were prepared with Alg, Gel, and MNP to serve as reservoirs of CAF. The effects of a magnetic stimulus and Ca^2+^ on the release of CAF were systematically investigated. Understanding the interactions between CAF and Ca^2+^, which have been scarcely reported in the literature, is also important because they might be associated with low calcium intake and skeletal fragility [33]. In this study, the effects of CAF release from Gel/Alg-based magnetic hydrogels in the absence and presence of Ca^2+^ on the viability of SH-SY5Y neuroblastoma cells were evaluated. Gel/Alg-based hydrogels capable of delivering adequate amounts of CAF may have medical applications and provide an avenue for cell viability improvement. 

## 2. Materials and Methods

### 2.1. Materials

Alginic acid sodium salt (Alg, Sigma 180947, mannuronate/guluronate ratio = 1.56, M_v_ ~ 150,000 g mol^−1^); gelatin pork skin (Gel, type A, Sigma G9382); FeCl_2_·4H_2_O, FeCl_3_·6H_2_O, and ammonium hydroxide (LabSynth, Diadema, Brazil); glutaraldehyde (GTA, Sigma G5882); calcium chloride (CaCl_2_, LabSynth); potassium chloride (KCl, LabSynth); and caffeine (CAF, Sigma C0750, 194.19 g mol^−1^) were used as received. All reagents were of analytical grade.

### 2.2. Preparation and Characterization of Magnetic Nanoparticles (MNP)

The synthesis of magnetic nanoparticles (MNP) was carried out by co-precipitation, as described elsewhere [34]. Briefly, 0.05 M FeCl_2_∙4H_2_O and 0.1 M FeCl_3_∙6H_2_O solutions were mixed in a 1:1 ratio (*v*/*v*). Then, ammonium hydroxide (25%, *v*/*v*) (a precipitating agent) was added dropwise to the mixture under vigorous stirring at room temperature up to pH 10. Then, the sonotrode MS7 (acoustic power density of 130 Wcm^−2^) coupled with the ultrasonic processor Hielscher UP100H (Hielscher Ultrasonics GmbH, Teltow, Germany) was immersed in the reaction flask at (24 ± 1) °C for 10 min. Afterward, rinsing steps were performed to remove excess reactants.

The crystalline structure of the oven-dried MNP was analyzed by X-ray diffraction (XRD, Bruker D2 Phaser-Germany) using CuK-alpha radiation source at 30 kV and 14 mA and in a 2θ range from 20 to 80° with a scan step of 0.02°. The mean size of MNP was determined by atomic force microscopy (AFM) using a Bruker multimode 8 AFM in Peakforce tapping mode in an air atmosphere. The probe used was a Si_3_N_4_ tip, with a spring constant of 0.4 N m^−1^. Image analysis was performed using the NanoScope 2.0 software. The MNP dispersion was diluted at a 1:100 ratio in ethanol. Then, the dispersion was deposited on Si/SiO_2_ wafers by spin coating at 3500 rpm. 

### 2.3. Preparation of Magneto-Responsive Hydrogels 

**Figure 1:**
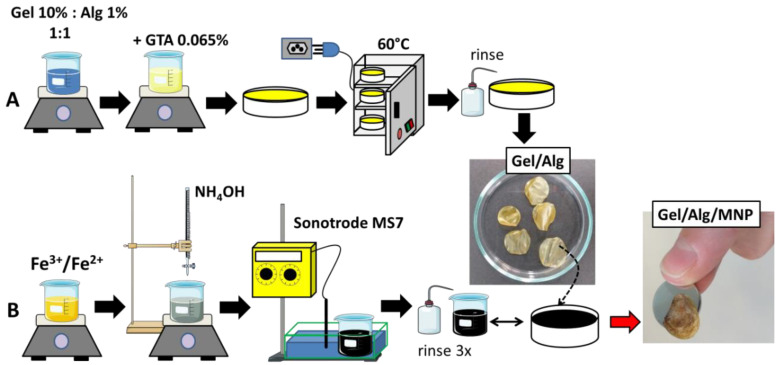
Synthesis of magneto-responsive hydrogels, Gel/Alg/MNP, using the blending method [14]. (**A**) Preparation of Gel/Alg hydrogels and (**B**) immersion of Gel/Alg dry films into MNP dispersion prepared by co-precipitation.

First, aqueous solutions of Alg at 1% (*w*/*v*) and Gel at 10% (*w*/*v*) were prepared separately by dissolving both substances in Milli-Q^®^ water. Alg solution at 1% was added to the Gel solution at 10% in a 1:1 ratio (Gel:Alg *v*/*v*) under magnetic stirring at 50 ± 2 °C for 40 min. Subsequently, GTA (crosslinker) was added at 0.065% (*w*/*v*). The mixture was homogenized and centrifuged at 3600 rpm (870 g) for 3 min to remove air bubbles. Then, the mixture was cast in polytetrafluoroethylene molds (3.5 cm diameter) and allowed to dry in an oven at 60 ± 2 °C overnight, as depicted in Figure 1A. After drying, the films were rinsed with Milli-Q water to remove the unreacted GTA molecules. The hydrogels were cut as disks of 1.6 cm diameter and coded as Gel/Alg. Magneto-responsive hydrogels were prepared by immersing dry Gel/Alg films in the MNP dispersion at pH 5.5 for 1 min followed by three rinses in Milli-Q water to remove excess MNP (Figure 1B). The magnetic scaffolds were coded as Gel/Alg/MNP.

### 2.4. Loading and In Vitro Release of CAF in the Absence and Presence of CME 

Dry disks of Gel/Alg and Gel/Alg/MNP were immersed into 10 mL of 5.0 g L^−1^ CAF aqueous solution for 1 h at 24 ± 1 °C. Then, Gel/Alg/CAF and Gel/Alg/MNP/CAF were rinsed with Milli-Q water to remove excess caffeine and dried in an oven at 60 °C overnight. Subsequently, the amount of loaded CAF (wt%) was quantified by elemental analysis with a Perkin-Elmer CHN 2400 device. The % N was determined for Gel/Alg, Gel/Alg/CAF, Gel/Alg/MNP, and Gel/Alg/MNP/CAF. To compare the increase in % N in Gel/Alg/CAF and Gel/Alg/MNP/CAF, pristine materials were added along with CAF, which contains 28.8% N. The loading efficiency (*LE*) (Equation (1)) of CAF was estimated by dividing the number of CAF mols found in the hydrogels (*n_CAF_*) by the number of CAF mols in the initial solution (*n_i_*) (5.0 g L^−1^):

(1) $$LE=\left(\frac{n_{CAF}}{n_{i}}\right)\times 100\%$$

The in vitro release of CAF was systematically evaluated in the absence and presence of an EMF (0.4 T) by immersing the CAF-loaded discs into 3 mL of different media/conditions: (i) at pH 5.5 (MilliQ^®^ water) and 25 °C; (ii) at pH 5.5 (MilliQ^®^ water) and 37 °C; (iii) in CaCl_2_ solution (0.5 M) at pH 5.5 and 25 °C; (iv) at pH 7.4 (PBS) and 37 °C; and (v) in CaCl_2_ solution (0.002 M) at pH 7.4 (PBS) and 37 °C, mimicking plasma conditions. The release of CAF was monitored over 360 min in the absence and presence of an EMF generated by NdFeB magnets with a magnetic field strength of 0.4 T, as shown in Figure S1. At intervals of 15 min, a 1.5 mL aliquot was withdrawn and replaced by 1.5 mL of MilliQ water (pH 5.5), PBS buffer (pH 7.4), PBS/CaCl_2_ at pH 7.4 (0.002 M), or CaCl_2_ (0.5 M, pH 5.5) solution to complete the initial volume. The concentration of CAF released in each withdrawn aliquot was evaluated using the calibration curve at 272 nm, which is the wavelength of maximal caffeine absorbance (Figure S2).

### 2.5. Characterization 

The gel content (GC), swelling degree (SD), and chemical stability in the pH range 2–12 of Gel/Alg and Gel/Alg/MNP hydrogels were evaluated at equilibrium after three drying–wetting cycles according to Equations (2) and (3), respectively:

(2) $$SD=\left(\frac{m_{SW}-m_{i}}{m_{i}}\right)\times 100$$

(3) $$GC=\left(\frac{m_{s}}{m_{i}}\right)\times 100$$

where *m_i_* is the initial mass of dried polymer, *m_SW_* is the mass of the swollen hydrogel, and *m_s_* is the mass of dried hydrogel after extensive rinsing to remove the unreacted molecules.

The morphologies of Gel/Alg, Gel/Alg/CAF, Gel/Alg/MNP, and Gel/Alg/MNP/CAF were evaluated using a JEOL Neoscope JCM-5000 microscope at 15 kV. Freeze-dried hydrogels were cryo-fractured, and their surfaces were coated with a 2 nm-thick sputtered gold layer before the analyses. Fourier transform infrared vibrational spectroscopy in the attenuated total reflectance mode (FTIR-ATR) was applied using a Perkin Elmer Frontier equipment, and a ZnSe crystal, in the wavenumber range of 600–4000 cm^−1^ with a resolution of 4 cm^−1^. FTIR spectra of Gel, Alg, and CAF were obtained using KBr pellets.

The iron content in the magnetic hydrogels (Gel/Alg/MNP and Gel/Alg/MNP/CAF) was determined by Flame Atomic Absorption Spectroscopy (FAAS—Vario 6) with an iron hollow-cathode lamp, wavelength of 248 nm, deuterium background correction lamp, lamp current of 5 mA, and fuel Acetylene/Air. The results allowed for the calculation of the Fe_3_O_4_ content in the samples. The superparamagnetic properties of Gel/Alg/MNP and Gel/Alg/MNP/CAF samples were revealed with a LakeShore 7404 vibrating sample magnetometer at room temperature (25 °C) and an external field in a range of −15 kOe to 15 kOe. 

The mechanical properties of dried hydrogels were evaluated using a DMA Q800 (TA Instruments) for samples ~ 0.300 mm thick with rectangular dimensions (6 mm × 10 mm between the grips) after they were dried at 50 °C until attaining a constant weight and stored in a desiccator. They were removed from the desiccator just before the tensile strength tests, which were performed, for five samples of the same type, at 30.0 ± 1.0 °C, under a N_2_ atmosphere, and at 1.00 N min^−1^. Thermogravimetric analyses (TGA) were performed in a TGA Q500 (TA Instruments) system. Pt crucibles containing the samples (typically 10 mg) were heated at a rate of 15 °C min^−1^, ranging from 30 °C to 500 °C, under N_2_ atmosphere (50 mL min^−1^).

### 2.6. Calcium Ion Diffusion through the Hydrogels

The permeation of Ca^2+^ through the hydrogels in the presence and absence of CAF and MNP was investigated using a potentiometric microsensor. The electrochemical experiments were carried out with an Autolab PGSTAT128N bipotentiostat (Metrohm Autolab, Utrecht, The Netherlands) coupled with a Sensolytics (Sensolytics, Bochum, Germany) SECM workstation. The measurements were performed using an ion-selective liquid-membrane microelectrode (ISME-Ca^2+^) and Ag/AgCl (KClsat) as the indicator and reference electrodes, respectively. The ISME-Ca^2+^ was fabricated according to a methodology previously described in the literature [35,36]. Therefore, the tip of a clean borosilicate micropipette manufactured in a P-2000 laser-based micropipette puller system (Sutter Instrument Co., Novato, CA, USA) was silanized, and the ionophore membrane (ETH 1001, Sigma Aldrich) was inserted by capillarity. Then, using a flexible syringe (MicrofilTM 34G, WPI Inc., Sarasota, FL, USA), the micropipette was filled with a 100 mM CaCl_2_ reference solution. An Ag|AgCl wire was added to the set to complete the indicator electrode system and provide electrical contact. The ISME-Ca^2+^ was stored in a 50 mM CaCl_2_ solution for 1 day before use. The potentiometric response was obtained from open circuit potential (OCP) measurements and evaluated using calibration curves obtained in 0.1 M KCl solution (pH 7) containing CaCl_2_ at different concentrations starting from 0.1 µM to 0.3 M (0.5 ≤ pCa^2+^ ≤ 7). To ensure data integrity, calibration curves were determined before and after the use of the sensor during a working day. A two-compartment electrochemical cell was used to evaluate the diffusion of Ca^2+^ through the scaffolds, and the positioning system of an SECM (Scanning Electrochemical Microscopy) device allowed for the precise positioning of the ISME-Ca^2+^. The experimental setup (Figure S3) enabled the use of two different solutions in each compartment: one with a 0.1 M KCl solution (pH 7) containing 0.5 M CaCl_2_ (lower compartment) and the other with a 0.1 M KCl solution (pH 7) (upper compartment). The potentiometric sensor was then positioned 300 µm away from the scaffold surface [36,37], and Ca^2+^ was allowed to diffuse from the lower compartment to the upper one only through the scaffold. Changes in Ca^2+^ concentration in the upper compartment were monitored by OCP measurements. 

In order to assess the influence of Ca^2+^ flow on the CAF release through the hydrogels, the Gel/Alg/CAF film was positioned between the upper and lower compartments of the electrochemical cell. Then, a 0.1 M KCl solution was added to the upper and lower compartments, and the concentration of CAF released to both compartments was measured in the presence and absence of CaCl_2_ (0.5 M) flow via UV spectrophotometry (272 nm).

### 2.7. Cell Viability 

The SH-SY5Y human neuroblastoma cell line (American Type Culture Collection ATCC CRL-2266) is a suitable model for investigating neuronal diseases because SH-SY5Y cells differentiate into dopaminergic, cholinergic, and adrenergic neuronal phenotypes. Some advantages related to SH-SY5Y in vitro studies compared to the use of primary neurons include their large-scale growth, easy manipulation, low cost, morphological similarity to primary neurons, and ability to be conducted without ethical approval, which would be necessary for primary cell culture [38,39].

SH-SY5Y neuroblastoma cells were grown in cell-culture-grade flasks (75 cm²) containing Dulbecco’s Modified Eagle Medium (DMEM/F-12) supplemented with 1 mM of CaCl_2_, 10% fetal bovine serum (FBS), and 1% streptomycin/penicillin at 37 °C, 5% CO_2_, and 95% humidity according to the ATCC (American Type Culture Collection) protocol. At 80% confluence, the cells were detached enzymatically with TrypLE (Thermofisher Scientific) and seeded into an adherent plate. 

First, a dose–response curve was created to evaluate the effects of increasing CAF concentrations on the viability of SH-SY5Y cells. The cells were seeded at 1 × 10^5^ cells mL^−1^ in a 48-well plate. After 24 h, the cells were incubated with CAF in the concentration range from 0.2 mM to 100 mM in DMEM/F-12 without supplements. After 24 h, metabolic activities as measures of cell viability rates were determined by the MTT (3-(4,5-dimethyl-2-thiazolyl)-2,5-diphenyl-2H-tetrazolium bromide) assay. MTT solution at 0.3 mg mL^−1^ final concentration was added to the cells. After 3 h, the MTT solution was extracted, and dimethyl sulfoxide was added to solubilize the formazan product, which had purple coloration. The increase in the intensity of the purple color was related to an increase in cell number or their metabolic activity. The absorbance (Optical Density—OD) measured at 570 nm (corresponding to the purple color) in a microplate reader (FlexStation 3, Molecular Devices, San Jose, CA, USA) allowed for the estimation of cell viability:

(4) $$Cell\;viability\;\left(\%\right)=\frac{OD_{s}-OD_{B}}{OD_{c}-OD_{B}}$$

where *OD_S_* = optical density of the sample (cells incubated with CAF and DMEM/F-12 medium); *OD_C_* = the optical density of the control (cells incubated only with DMEM/F-12 medium); and *OD_B_* is the optical density of blank (incubation without cells).

In order to evaluate the effect of CAF release from magnetic hydrogels on the viability of neuroblastoma cells, in vitro cytotoxicity assays were performed using the solution extract and according to ISO 10993-5:2009 (International Organization for Standardization, 2019) protocol. Prior to use, the hydrogels were sterilized by exposure to UV radiation. Then, SH-SY5Y cells were seeded in a 48-well plate at 1 × 10^5^ cells mL^−1^. Simultaneously, hydrogels were placed into a 24-well plate with 1 mL of supplemented DMEM/F-12 medium in the absence of an EMF. After 24 h, cells were treated with 250 µL of the extract solution stemming from the hydrogels. Then, after 24 h and 48 h of treatment, the MTT assay was performed, and the cell viability was calculated according to the Equation (4).

### 2.8. Statistical Analysis 

Experimental results were expressed as the mean value and the corresponding standard deviation (mean ± SD). All data were analyzed by ANOVA (one-way analysis of variance) to assess differences among the groups and *p* values < 0.05 were classified as statistically significant. At least three independent experiments were carried out for each assay.

## 3. Results and Discussion

### 3.1. Characterization of MNP

The concentration of magnetite in the dispersion, which was determined via gravimetric analysis, was 5 ± 1 g L^−1^. The XRD pattern of MNP, shown in Figure S4, showed peaks at 2ϴ 30.2°, 35.7°, 43.3°, 53.8°, 57.2°, 62.9°, and 74.5°, which were attributed to the diffraction planes at (220), (311), (400), (422), (511), (440), and (533), respectively, indicating the presence of cubic spinel phase of Fe_3_O_4_ [40,41,42]. Peaks attributed to maghemite (ϒ-Fe_2_O_3_) ((113) (210)) and hematite (ɑ-Fe_2_O_3_) ((104) (024)) [40,43] and peaks from other crystalline phases were not identified. Using the Scherrer equation (Equation (5)), the mean size of the crystalline domain was determined to be 10.77 nm, for which the highest intensity peak was considered, which corresponded to the (311) plane [44]:

(5) $$d=\frac{K\lambda }{\beta \;cosϴ}$$

where *λ* is the wavelength of radiation (Cu kα 0.154 nm); *β* is the full width at half maximum (311 peak); 2*ϴ* is the diffraction angle (Bragg angle); and *K* is the particle shape factor (0.94).

AFM topographic images (Figure S5a,b) allowed for the estimation of the mean size of the MNP. A total of 106 particles were randomly chosen and a transverse line was drawn on each nanoparticle to assess the height of the particles, as the height is equal to the diameter in spherical particles. According to the size distribution shown in Figure S5c, the MNP presented an average diameter of 3.1 ± 1.5 nm. MNP with very small size, below 20–50 nm, are classified as superparamagnetic particles [14,41,45]. The MNP size obtained from the AFM images was smaller than the size obtained from XRD data calculated with the Scherrer equation (~11 nm). The samples for AFM were spin-coated. Upon spinning, it is possible that the larger particles were removed together with the solvent and that only the very small ones remained on the surface.

### 3.2. Characterization of Gel/Alg and Gel/Alg/MNP Hydrogels 

**Figure 2:**
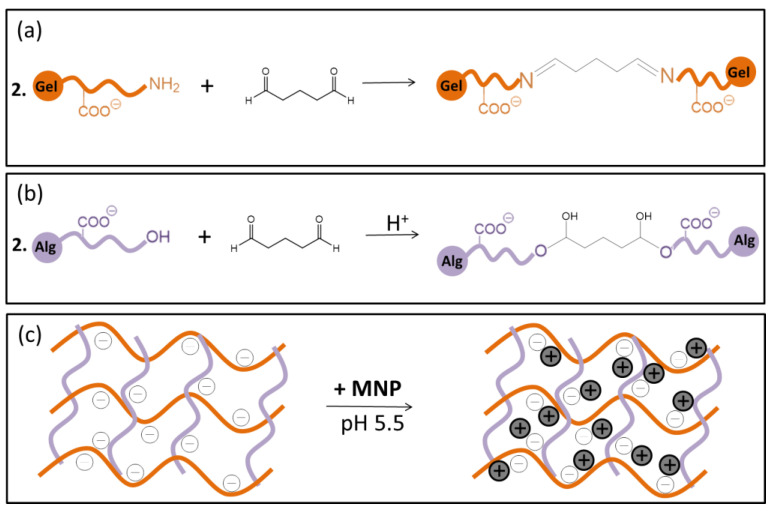
Representation of the crosslinking reaction of Gel/Alg hydrogels with glutaraldehyde via (**a**) base Schiff formation between Gel amino groups and GTA aldehyde and (**b**) the formation of acetal bonds between Alg hydroxyl groups and GTA aldehyde groups in acidic medium. (**c**) After crosslinking, MNP were loaded into the hydrogels via electrostatic interactions between carboxylate groups and positively charged MNP.

The crosslinking of Gel chains by glutaraldehyde (GTA) transpired via Schiff’s base formation [46], as schematically depicted in Figure 2a. The reaction was carried out under slightly acidic conditions (pH 5.0). The GTA aldehyde groups can also react with Alg hydroxyl groups, forming hemiacetal, which can further react with another hydroxyl group, forming acetal groups, as schematically represented in Figure 2b. 

**Figure 3:**
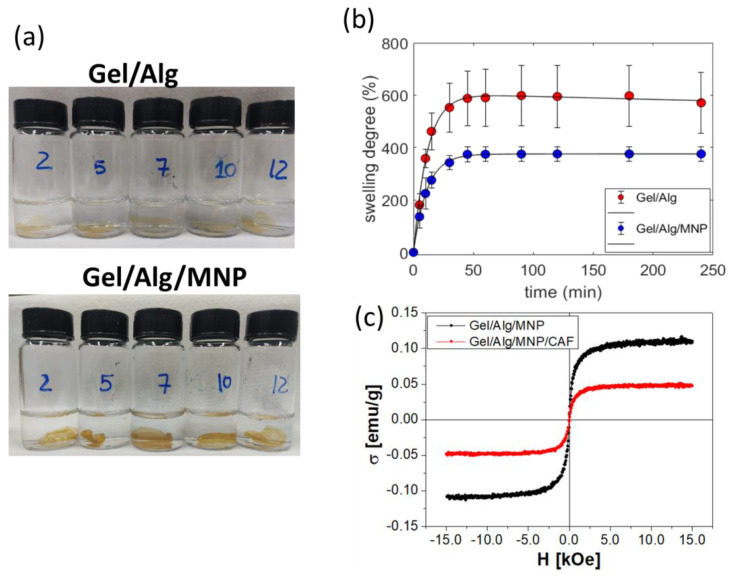
Stability tests of (**a**) Gel/Alg and Gel/Alg/MNP over 30 days of exposure in the pH range from 2 to 12. (**b**) Swelling degree of the Gel/Alg and Gel/Alg/MNP hydrogels in Milli-Q water at 25 °C as a function of time; the solid lines serve as a guide for the eyes. (**c**) Hysteresis loops obtained by VSM for the magnetic hydrogels in the absence (black) and presence (red) of caffeine.

Gel/Alg and Gel/Alg/MNP presented outstanding chemical stability in the pH range of 2–12, as shown in Figure 3a, because the crosslinking reaction with GTA molecules produced covalent bonds and stable linkages. Figure 3b shows the swelling degree (SD) of the Gel/Alg and Gel/Alg/MNP hydrogels in Milli-Q water at 25 °C as a function of time. Equilibrium was achieved after 50 min; the equilibrium SD values of Gel/Alg and Gel/Alg/MNP hydrogels amounted to 570 ± 117% and 376 ± 27%, respectively. The gel content (GC) values determined for Gel/Alg and Gel/Alg/MNP were 83.4 ± 2.3% and 93.6 ± 1.2%, respectively. Noteworthily, Gel/Alg/MNP presented higher GC (*p* < 0.05) and lower SD values (*p* < 0.05) than Gel/Alg, indicating that the MNP contributed to the building of networks between the macromolecules. At pH 5.5, the alginate and gelatin carboxylic acid groups were deprotonated, and the MNP were positively charged; the MNP have an isoelectric point at pH 6 [47]. Thus, electrostatic interactions drive the interactions between the macromolecules and MNP (Figure 2c). 

The iron content analysis showed 0.22 wt% of Fe in the magnetic hydrogels, which corresponds to 0.3 wt% of Fe_3_O_4_. According to Figure 3c, the MNP incorporated into Gel/Alg/MNP presented superparamagnetic behavior (no coercivity) and saturation magnetization of 0.11 emu g^−1^ at 300 K. Magnetic alginate beads presented similar magnetization characteristics [30].

**Figure 4:**
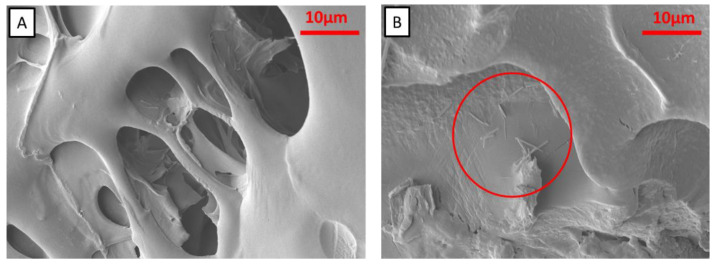
SEM images of hydrogels (**A**) before (Gel/Alg/MNP) and (**B**) after caffeine incorporation (Gel/Alg/MNP/CAF). The red circles indicate the caffeine needles.

Scanning electron microscopy (SEM) analyses indicated irregular macroporous structures in Gel/Alg (Figure S6A) and Gel/Alg/MNP (Figure 4A). Gel, Alg, Gel/Alg, and Gel/Alg/MNP FTIR presented similar spectra (Figure S7a). The imine bands related to Schiff base formation and the characteristic alginate bands overlapped the bands characteristic of pure gelatin. The FTIR spectra in Figures S7a,b show an absorption band at 3300 cm^−1^, which was attributed to the N-H stretching of amine and O-H stretching, and the small peak at 2950 cm^−1^ was ascribed to the stretching of C-N and N-H, corresponding to the gelatin amines or the C-H stretching of alginate. The bands at 1630 cm^−1^ and 1530 cm^−1^ were attributed to the C=O stretching of amide I and the N-H stretching of amide II, respectively. Moreover, the bands at 1240 cm^−1^ were ascribed to the C-N and N-H stretching of amine, and the band at 1077 cm^−1^ was attributed to C-N axial deformation [48,49].

CHN elemental analysis of nitrogen indicated 1.54 wt% (15.4 mg/g) and 1.85 wt% (18.5 mg/g) of caffeine (CAF) incorporated into the Gel/Alg and Gel/Alg/MNP hydrogels, whereas the loading efficiency values compared to the initial concentration of CAF amounted to 0.66% and 0.72%, respectively. The loading of CAF into Gel/Alg and Gel/Alg/MNP was evidenced by the presence of caffeine crystals (needles) [50] on the surface (Figure 4B and Figure S6B). FTIR spectra showed a weak band at 760 cm^−1^, which was only identified in the spectra of pure CAF, Gel/Alg/CAF, and Gel/Alg/MNP/CAF (Figure S7b,c); it was assigned to the bending vibrations outside the plane of the carbonyl group (C=O) of caffeine [51]. Furthermore, this band was absent in the spectra of the caffeine-free hydrogels (Gel/Alg and Gel/Alg/MNP), Gel, and Alg. The magnetization saturation of Gel/Alg/MNP/CAF was about half of that measured for Ge/Alg/MNP (Figure 3c); this phenomenon might be due to the leaching of weakly attached MNP during the incorporation of CAF. 

Figure S8 presents the typical stress–strain curves determined for the dried Gel/Alg, Gel/Alg/CAF, Gel/Alg/MNP, and Gel/Alg/MNP/CAF hydrogels; the mean values of Young’s modulus (E) amounted to 2889 ± 275 MPa, 2466 ± 306 MPa, 2216 ± 155 MPa, and 2187 ± 257 MPa, respectively. No statistically significant difference (*p* > 0.05) among the E values was observed for the Gel/Alg-based dried hydrogels. For comparison, the E values reported for dried Gel/Alg-based hydrogels crosslinked with Ca^2+^ ions ranged from ~1000 MPa to 1200 MPa [52], indicating that glutaraldehyde led to stiffer Gel/Alg networks than those prepared by gelation with Ca^2+^ ions. 

The thermal properties of the hydrogel samples were investigated using thermogravimetric analysis. The TG/DTG curves are provided in the Supplementary Materials (SM9). All samples presented a mass loss in the range from 25 °C to 150 °C due to the release of water molecules, which ranged from 11.5 wt% to 6.9 wt% (Table 1). All samples also presented a mass loss of ~60 wt% in the range from 150 °C to 500 °C due to the decomposition of alginate [30,53] and gelatin [54,55]. The main decomposition peaks (T_dec_) appeared in the temperature range of 320 °C to 340 °C (Table 1). The temperature at which 50% of the original mass was lost (T_50%_) ranged from 337 °C to 342 °C. In comparison to Gel/Alg, the addition of CAF, MNP, or both did not significantly affect the thermal stability of the hydrogels, which was probably due to their low content in the hydrogels. Gel/Alg/CAF (Figure S9b) showed a peak at 226 ± 1 °C, which was attributed to the melting of CAF [50], and a peak at 448 ± 1 °C, which might be due to the conversion of alginate into Na_2_CO_3_ [55]. Gel/Alg/MNP (Figure S9c) and Gel/Alg/MNP/CAF (Figure S9d) showed a peak at 210 ± 1 °C, which was ascribed to the onset of the degradation of Fe_3_O_4_ [56].

### 3.3. Diffusion of Ca^2+^ Ions through the Hydrogels

Calcium ions are involved in intra- and extracellular activities, including the regulation of metabolic and apoptotic activity, cell adhesion, charge carriers, and intracellular messengers. In neuronal cells, calcium ions play an important role in synaptic transmission [57,58]. The action potential arrives in the axon terminal and triggers the calcium ion channels, which causes voltage-gated calcium channels in the active zones to open. The increase in the concentration of intracellular calcium ions leads to synaptic vesicle exocytosis; consequently, neurotransmitters can be released from synaptic vesicles to the postsynaptic neuron and bind to specific receptors [59,60,61]. These mechanisms enable the proper functioning of synaptic transmission between neurons. Therefore, a scaffold for drug delivery or the tissue engineering of neuronal cells should allow for efficient calcium ion diffusion. For this reason, Ca^2+^ transport through the hydrogels was evaluated using potentiometric measurements.

To conduct a potentiometric analysis of Ca^2+^ diffusion through the hydrogels, a homemade cell of poly(methyl methacrylate) combined with a bipotentiostat and an SECM workstation was used inside a Faraday cage. This system is schematically represented in Figure S3. The hydrogel was placed between the upper and lower compartments. In this setup, the permeation of Ca^2+^ ions from the lower compartment to the upper one only takes place through the hydrogel. When calcium ions diffuse across the hydrogel and reach the upper compartment, the concentration of calcium ions is locally measured by an ISME-Ca^2+^ microelectrode. Figure S10a shows the results of a typical experiment, and a linear potential increase is observed upon increasing the Ca^2+^ ion concentration starting from 10 µM (pCa^2+^ = 5). A Nernstian response can be observed on the curves recorded before and after using the sensor, which possess very similar slopes (26.1 and 25.9 mV pCa^2+^ unit^−1^), indicating that the ISME-Ca^2+^ sensor’s sensitivity does not change during long-term operation. 

**Figure 5:**
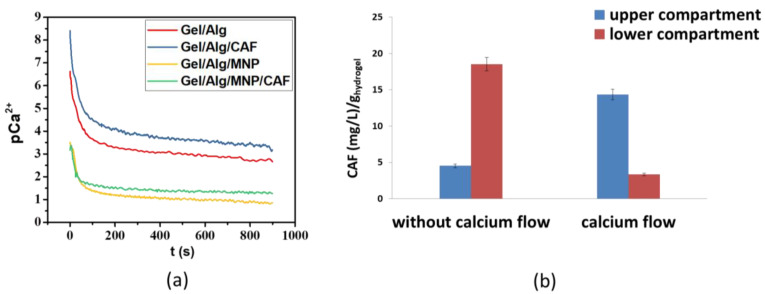
(**a**) Ca^2+^ ion permeation through Gel/Alg, Gel/Alg/CAF, Gel/Alg/MNP, and Gel/Alg/MNP/CAF hydrogels; (**b**) influence of Ca^2+^ ion (0.5 M) flow on the concentration of CAF released normalized by the hydrogel mass.

The permeation of Ca^2+^ ions was investigated for the Gel/Alg, Gel/Alg/CAF, Gel/Alg/MNP, and Gel/Alg/MNP/CAF hydrogels, whose mean thickness value was 500 ± 50 µm. OCP measurements were recorded for 900 s. Figure 5a presents the changes in the concentration of Ca^2+^ ions that diffused through the hydrogels to the upper compartment over 15 min. One should note that pCa^2+^ = −log [Ca^2+^]; thus, the smaller the value of pCa^2+^, the higher the Ca^2+^ concentration. In comparison to the Gel/Alg and Gel/Alg/CAF hydrogels, Ca^2+^ ions permeated more pronouncedly through their magnetic counterparts (Gel/Alg/MNP and Gel/Alg/MNP/CAF). The presence of MNP in the polymeric matrix can cause the elongation or compression of the chains due to the orientation of the dipoles. Such magneto-mechano effects can stimulate the diffusion of Ca^2+^ through the hydrogels [36]. These results are consistent with those of previously reported studies, in which the presence of MNP in scaffolds favored cell growth due to an enhanced influx of calcium ions [19,36].

Comparing the curves for Gel/Alg and Gel/Alg/CAF in Figure 5a, it is clear that the presence of CAF in the hydrogels caused a slight decrease in Ca^2+^ ion permeation. One hypothesis in this regard is that favorable interactions between CAF and Ca^2+^ ions can reduce the Ca^2+^ ions’ diffusion and CAF release from the hydrogels. This was examined by determining the amount of caffeine present in both compartments under two different conditions: with and without Ca^2+^ ion flow for 15 min. The CAF concentration was determined by UV spectrophotometry (272 nm). Figure 5b shows that caffeine is released to both compartments under both conditions. However, in the absence of the flow of Ca^2+^ ions, CAF was released in higher quantities to the lower compartment, whereas the opposite was observed in the presence of the flow of Ca^2+^ ions. A plausible explanation for such an observation is that Ca^2+^ ion permeation favored the release of caffeine into the upper compartment because of the chelation of Ca^2+^ ions by the CAF molecules [62]. Ca^2+^ ions interact with the isolated carbonyl of caffeine, and divalent cations interact favorably with the negative dipole of CAF (carbonyl group O6) [63,64]. This kind of interaction explains the decreased mobility of the Ca^2+^ ions through the CAF-loaded hydrogels. 

The fact that CAF was detected in the compartment containing the ISME-Ca^2+^ sensor, even in the absence of Ca^2+^ flow, raised the question of whether CAF has any influence on the sensor’s signal. Therefore, OCP measurements were recorded in a 0.1 M KCl solution, with and without 1 mM of CaCl_2_, in the presence of CAF. The addition of CAF did not significantly change the potential measured with the ISME-Ca^2+^ sensor (Figure S10b) in the presence or absence of Ca^2+^ ions. The slight fluctuations in potential can be attributed to the addition of the CAF stock solution to the medium. Therefore, it can be concluded that CAF did not cause any interference in the sensor’s response during the study of Ca^2+^ ion permeation. The effect of calcium ions on the CAF release kinetics from the hydrogels is presented in Section 3.4.

### 3.4. In Vitro Release of Caffeine

Kinetic models help describe the release mechanism of a drug from a matrix. Different models (such as the zero-order, first-order, Higuchi, Hixson–Crowell, Korsmeyer–Peppas, Baker, Lonsdale, Weibull, Hopfenberg, and Cooney models) consider various effects such as matrix dissolution, swelling, erosion, and drug diffusion [65]. The Higuchi and Korsmeyer–Peppas models are commonly applied to describe drug delivery from polymers and hydrogels [30,46,53,54]. According to Higuchi’s model, a drug diffuses freely from a porous matrix to a medium in accordance with Fick’s law, and matrix swelling is negligible, whereas Korsmeyer–Peppas’s model concerns a drug’s transport through a swellable matrix [66,67,68]. CAF was incorporated into Gel/Alg matrices, which are porous (Figure 4) and swellable (Figure 3b). For this reason, the experimental data on drug release were fitted with the Higuchi and Korsmeyer–Peppas models.

The in vitro release of CAF from the hydrogels was evaluated in the presence (Gel/Alg /MNP/EMF) and absence (Gel/Alg, Gel/Alg/MNP) of an EMF under five different conditions: (i) at pH 5.5 and 25 °C; (ii) at pH 5.5 and 37 °C; (iii) in CaCl_2_ 0.5 M at pH 5.5 and 25 °C; (iv) at pH 7.4 (PBS) and 37 °C; and (v) in CaCl_2_ solution (0.002 M) at pH 7.4 (PBS) and 37 °C, mimicking plasma conditions. Figure S11a–e show the cumulative CAF release normalized by the hydrogel mass over 360 min. The experimental data were fitted using the Korsmeyer–Peppas model (Equation (6)) [69] and Higuchi model (Equation (7)) [67]:

(6) $$\left(\frac{M_{t}}{M_{eq}}\right)=k_{KP}t^{n}$$

where *M_t_* is the released amount at time “*t*”, *M_eq_* is the released amount at equilibrium, *k_KP_* is a constant related to the release rate, and “*n*” is the diffusional coefficient, which describes the release mechanism and is related to the interactions between a drug and a matrix. When the solvent transport rate or diffusion is much greater than the process of polymeric chain relaxation, drug release is diffusion-controlled and *n* = 0.5 [70]. If *n* < 0.5, drug release is fast and the interactions between the drug–polymer-matrix are weak, whereas for 0.5 < *n* < 1.0, drug release is retarded because the rearrangement of polymeric chains occurs slowly, and the drug–polymer-matrix interactions are favorable [30,70].

(7) $$\;\;M_{t}=k_{H}\sqrt{t}$$

In the equation above, *k_H_* is a constant. The Higuchi model contends that release is solely controlled by the drug’s diffusion from the matrix to the solution.

**Figure 6:**
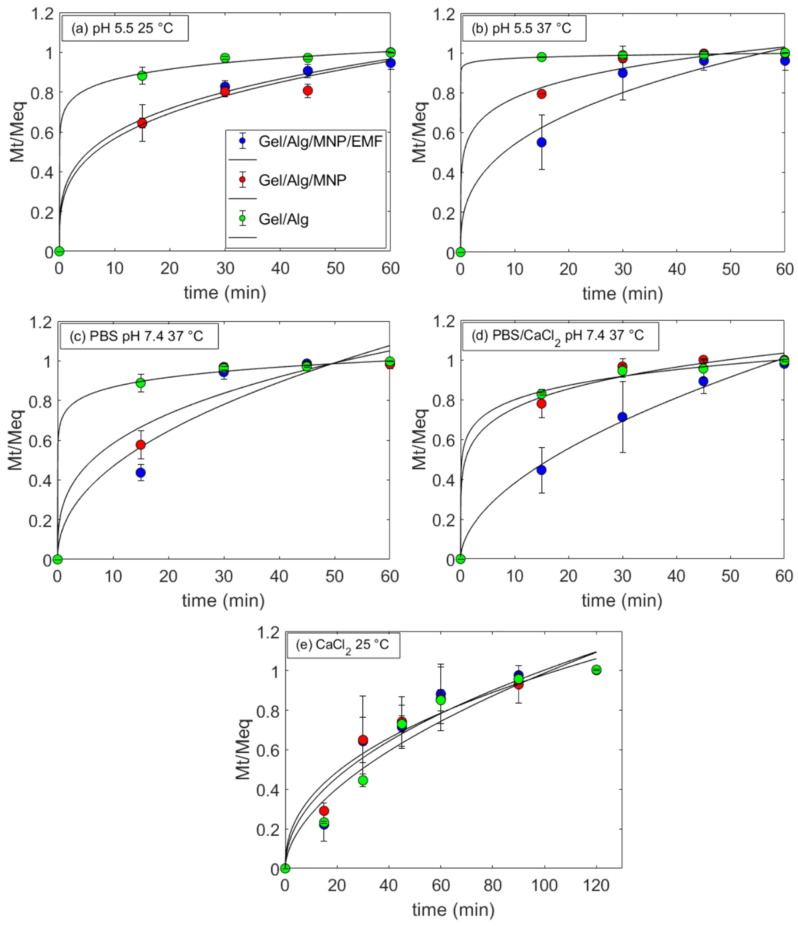
Caffeine release kinetics from the hydrogels in the absence and presence of MNP and EMF at (**a**) pH 5.5 (Milli-Q^®^ water) and 25 °C, (**b**) at pH 5.5 and 37 °C, (**c**) in PBS buffer and at 37 °C, (**d**) in PBS/CaCl_2_ (0.002 M) at 37 °C, and (**e**) in CaCl_2_ (0.5 M) at pH 5.5 and 25 °C, along with the corresponding non-linear degrees of fitting with respect to the Korsmeyer–Peppas model (solid lines).

Figure 6a–e show the cumulative CAF release along with the corresponding values of non-linear fitting with respect to the Korsmeyer–Peppas model; the values of non-linear fitting with respect to the Higuchi model are available in Figure S12a–e. Table 2 shows the fitting parameters. The correlation coefficient values (R^2^) indicated that the experimental data better fitted the Korsmeyer–Peppas model (R² > 0.93) than the Higuchi model. A plausible explanation for this is that the Higuchi model’s premises (negligible swelling or dissolution of the matrix and constant drug diffusivity) [70] do not hold for the systems investigated in this study. The Korsmeyer–Peppas model fitted better because the release of CAF to the solution depends on its diffusion through the swollen Gel/Alg hydrogels. 

The fitting parameters *k_KP_* and *n* presented in Table 2 for systems at pH 5.5 revealed that all *n* values were smaller than 0.5, which is typical of fast release. The statistical analysis carried out for the fitting parameters obtained from the Korsmeyer–Peppas model (Figure S13) clearly showed that the addition of MNP increased the degree of affinity between CAF and the matrix because *n* increased and *k_KP_* decreased, particularly at pH 5.5 at 25 °C, pH 5.5 at 37 °C, and in PBS at pH 7.4 and 37 °C (*p* < 0.05, *p* < 0.0005, and *p* < 0.05, respectively). This effect might be due to hydrogen bonds between CAF carbonyl groups and MNP hydroxyl groups, which decreased the release of CAF [51,54]. 

In the absence of an EMF, the release of CAF was favored upon increasing the temperature from 25 °C (Figure 6a) to 37 °C (Figure 6b) because *k_KP_* tended to increase and *n* tended to decrease. Upon increasing the temperature, the system´s thermal energy increased, and the caffeine–hydrogel interaction weakened [71,72,73].

At pH 7.4 and 37 °C (Figure 6c), a sustained release (*n* = 0.651) of CAF was observed for the magnetic hydrogels exposed to an EMF, and the *k_KP_* value (0.088 min^−1^) was almost one order of magnitude smaller than the values determined in the absence of an EMF or for the non-magnetic hydrogels. This effect was even more pronounced in PBS/CaCl_2_ (0.002 M) at 37 °C (Figure 6d), yielding *n* = 0.758 and a *k_KP_* value of 0.053 min^−1^ in the presence of an EMF. In the absence of an EMF, the n and *k_KP_* values amounted to 0.173 and 0.515 min^−1^. These findings clearly show that exposure to an EMF and the presence of Ca^2+^ ions in the medium retarded the release of CAF because the drug–polymer-matrix interactions became more favorable. The rearrangement of chains in the magnetic hydrogels might be decelerated under exposure to an EMF, and the influx of Ca^2+^ into the matrix might hinder the release of CAF due to the favorable interactions between CAF and Ca^2+^ ions (Figure 5). As shown in Figure S13b–d, the *k_KP_* and *n* values obtained for Gel/Alg/MNP/EMF were statically different than those determined for Gel/Alg/MNP at (i) pH 5.5 at 37 °C, (ii) in PBS pH 7.4 at 37 °C, and (iii) in PBS/CaCl_2_ at pH 7.4 and 37 °C.

The effect of Ca^2+^ ions was more dominant than that of an EMF. For instance, after 45 min, the released amount of CAF from Gel/Alg in PBS without Ca^2+^ ions amounted to 12.8 ± 0.2 mg/g_hydrogel_ (Figure S11c), whereas in PBS/CaCl_2_ (0.002 M) this value was 8.3 ± 0.2 mg/g_hydrogel_ (Figure S11d). At pH 5.5, the increase in the Ca^2+^ ion concentration to 0.5 M (Figure 6e) led to *n* values > 0.5, but without any statistically significant differences among them (Figure S13e). After 45 min, the amounts of CAF released from Gel/Alg, Gel/Alg/MNP, and Gel/Alg/MNP/EMF in PBS with 0.5 M Ca^2+^ ions were 5.7 ± 0.5 mg/g_hydrogel_, 5.0 ± 1.9 mg/g_hydrogel_, and 3.5 ± 1.4 mg/g_hydrogel_ (Figure S11e), respectively. 

The increase in medium ionic strength through the addition of CaCl_2_ also increased the potency of osmotic effects, thereby decelerating release. Addo and colleagues reported that drug delivery from HPMC matrices after 1 h at pH 1.2 was 30%, but it decreased to 26% when the ionic strength was increased to 0.4 M [74]. One reason for this result is that increasing ionic strength might decrease the swelling ratio [75]. However, the swelling degree values determined for Gel/Alg and Gel/Alg/MNP in Milli-Q water at 25 °C and 37 °C and in CaCl_2_ (0.5 M) at 25 °C were similar (Figure S14). 

Table 3 shows some examples of CAF carriers and their corresponding release behaviors. When polymer matrices are hydrophilic, the release of CAF tends to be fast (up to 30 min) because CAF is highly soluble in water (20 g L^−1^) [27,28,76,77]. Alginate-based blends and cellulose membrane loaded with CAF showed the release of approximately 50% and 80% of CAF after 5 min, respectively [27,77]. The burst release of CAF also transpired for nanofibers of PVA/caffeine within 60 s [76]. On the other hand, the CAF release from more hydrophobic matrices might take up to 48 h [50]. Caffeine release from hydrophilic matrices can be modulated by several factors, such as pH, temperature, and ionic strength, as reported in the literature [73,78,79,80,81]. However, there is scarce information about the combination of the application of a magnetic field (0.4 T) and Ca^2+^ ions in order to retard the CAF release from hydrophilic matrices, as demonstrated in this study. After 120 min, ~50% of total CAF was released from Gel/Alg/MNP in PBS/CaCl_2_ (0.002 M) at 37 °C. In comparison with other alginate-based matrices, the release behavior of CAF observed in this study was more sustained, but it was similar to that observed for alginate/CaCO_3_, which took 100 min [31]. The similarity between the CAF release time from Gel/Alg/MNP in PBS/CaCl_2_ (0.002 M) at 37 °C and the CAF release time from alginate/CaCO_3_ might be due to the interactions between CAF and Ca^2+^ ions, which are present in both systems. 

The literature reports suggest that the amount of CAF administered should be less than 60 mg over a period of 45 min to avoid the “crash effect” [5,6]. The amounts of CAF released from Gel/Alg/MNP in PBS/CaCl_2_ (0.002 M) at 37 °C (Figure S11d), at up to 45 min, and in the absence and presence of an EMF corresponded to 7.6 ± 0.4 mg/g_hydrogel_ and 6.1 ± 0.8 mg/g_hydrogel_, respectively. Under the same conditions, the release of CAF from non-magnetic Gel/Alg hydrogels was slightly higher (8.3 ± 0.2 mg/g_hydrogel_) and faster than those from Gel/Alg/MNP hydrogels. These findings disclose Gel/Alg-based hydrogels’ potential to serve as transdermal patches for the release of CAF, thereby circumventing the “crash effect”. 

### 3.5. Cell Viability

**Figure 7:**
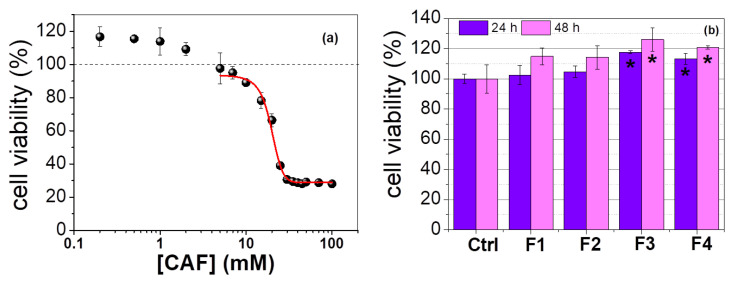
(**a**) Caffeine dose–viability response curve of SH-SY5Y cells treated with CAF in the concentration range from 0.2 mM to 100 mM over 24 h. The fit (red line) corresponds to y = 28.75 + (94.01 − 28.75)/(1 + 10^((19.67 − x)* − 0.1363)), for which R² = 0.99412. (**b**) Cell viability after the treatment with extracts from Gel/Alg (F1), Gel/Alg/MNP (F2), Ge/Alg/CAF (F3), and Gel/Alg/MNP/CAF (F4) hydrogels for 24 h and after 48 h incubation. Results are presented as mean values ± SD, for which * *p* < 0.05.

The administration of CAF in biological systems has been related to cell death [9,83,84,85] cell proliferation [86], and the prevention of apoptotic processes [11,87]. It seems ambiguous, but the effect of CAF on cell responses depends on its concentration, the type of cell, and the relevant experimental conditions [88]. For this reason, a dose–response curve was developed (Figure 7a) to evaluate the response of SH-SY5Y cells related to the CAF concentration after 24 h; Figure S15 shows the corresponding optical microscopy images. For CAF concentrations lower than 2 mM, the viability was higher than the control (without CAF). For CAF concentrations higher than 5 mM, the cell viability decreased. The dose–response fitting (Figure 7a, red line) allowed for the estimation of IC_50_ at 19.52 mM of CAF. These findings corroborate those from the literature, i.e., at 10 mM CAF, the enzymatic activity of caspase-3 might induce an apoptotic process [9], whereas in the concentration range between 10 and 100 μM CAF, cell apoptosis was prevented [11]. Sangaunchom and coworkers observed that 10 mM and 20 mM of CAF reduced the viability of SH-SY5Y to 75% and 34% of the untreated controls, respectively, after 24 h [89]. 

In order to evaluate the biocompatibility of magnetic and non-magnetic hydrogels, an MTT assay was performed using the hydrogel extracts in the absence of an EMF. Figure 7b indicates that the viability of the cells treated with the extracts was similar to or higher than that observed for the control. The cells treated with extracts from the Gel/Alg/CAF (F3) and Gel/Alg/MNP/CAF (F4) hydrogels exhibited a statistically significant increase in cell viability compared to the control. Notably, at pH 7.4 (PBS) + CaCl_2_ and at 37 °C, the Gel/Alg (F3) and Gel/Alg/MNP (F4) hydrogels released 0.34 mM and 0.37 mM of CAF, respectively. On the other hand, the viability of the cells treated with extracts from the Gel/Alg (F1) and Gel/Alg/MNP (F2) hydrogels was not significantly higher than the control due to the absence of CAF. These findings clearly show that (i) the amount of CAF delivered from Gel/Alg and Gel/Alg/MNP stimulated SH-SY5Y cells and that (ii) 2 mM of CAF is the maximal physiological concentration for stimulating SH-SY5Y cells, whereas higher concentrations promote their death.

## 4. Conclusions

This study demonstrated that the synergic effect of an EMF and Ca^2+^ ions led to a sustained release of CAF from Gel/Alg/MNP hydrogels. However, the effect of Ca^2+^ ions was more pronounced than the effect of an EMF due to the favorable interactions between CAF and Ca^2+^ ions, as evidenced by the potentiometric measurements. These findings might also be relevant for correlating CAF consumption and calcium balance in the human body. The amounts of CAF released from the Gel/Alg-based hydrogels in PBS/CaCl_2_ (0.002 M) at 37 °C for up to 45 min ranged from 6.1 ± 0.8 mg/g_hydrogel_ to 8.3 ± 0.2 mg/g_hydrogel_, thereby disclosing their potential to be applied as transdermal patches for the release of CAF at adequate levels. The amount of CAF released from the Gel/Alg and Gel/Alg/MNP hydrogels (~0.35 mM) increased the viability of SH-SY5Y cells, thus indicating their applicatory potential in tissue engineering. Thus, Gel/Alg and Gel/Alg/MNP hydrogels can be applied as suitable reservoirs of CAF when the final application requires the release of a low concentration of CAF to avoid adverse effects, such as the “crash effect”, or the achievement of improved neuronal cell proliferation.

## Figures and Tables

**Table 1:** Mean percentages of water loss (wt%) in the temperature range from 25 °C to 150 °C, decomposition peak (T_dec_), and temperature at which 50% of the original mass was lost (T_50%_) determined from the TG/DTG curves of Gel/Alg, Gel/Alg/CAF, Gel/Alg/MNP, and Gel/Alg/MNP/CAF samples (Supplementary Material Figure S9).

Sample	Water Loss (wt%)	T_dec_ (°C)	T_50%_ (°C)
Gel/Alg	11 ± 1	322 ± 1	337 ± 2
Gel/Alg/CAF	8.5 ± 0.8	322 ± 1	340 ± 1
Gel/Alg/MNP	8.5 ± 0.9	323 ± 2/340 ± 2	340 ± 1
Gel/Alg/MNP/CAF	6.9 ± 0.8	320.5 ± 0.8	342 ± 2

**Table 2:** Fitting parameters obtained for the release of CAF from Gel/Alg and Gel/Alg/MNP in the absence and presence of EMF. *k_KP_* (min^−n^) and *n* denote constant rate and diffusional coefficient (dimensionless) of Korsmeyer–Peppas model (Equation (6)). *k_H_* (mg of CAF/g of gel.min^−0.5^) denotes Higuchi’s constant rate (Equation (7)).

**Hydrogels**	**Korsmeyer-Peppas**			**Higuchi**	
	**pH 5.5 25 °C**				
	*k_KP_*	*n*	R^2^	*k_H_*	R^2^
Gel/Alg/MNP/EMF	0.316 ±0.024	0.278 ±0.018	0.997	1.213 ±0.061	0.947
Gel/Alg/MNP	0.298 ±0.113	0.294 ±0.096	0.987	2.179 ±0.004	0.949
Gel/Alg	0.714 ±0.095	0.084 ±0.036	0.998	2.092 ±0.007	0.811
	**pH 5.5 37 °C**				
	*k_KP_*	*N*	R^2^	*k_H_*	R^2^
Gel/Alg/MNP/EMF	0.238 ±0.060	0.361 ±0.013	0.970	1.928 ±0.079	0.951
Gel/Alg/MNP	0.537 ±0.009	0.159 ±0.003	0.993	1.953 ±0.065	0.871
Gel/Alg	0.943 ±0.001	0.014 ±0.002	0.999	2.019 ±0.008	0.738
	**PBS pH 7.4 37 °C**				
	*k_KP_*	*N*	R^2^	*k_H_*	R^2^
Gel/Alg/MNP/EMF	0.089 ±0.016	0.651 ±0.055	0.931	1.684 ±0.177	0.930
Gel/Alg/MNP	0.279 ±0.072	0.327 ±0.072	0.958	1.954 ±0.084	0.931
Gel/Alg	0.728 ±0.101	0.079 ±0.036	0.999	2.044 ±0.126	0.808
	**PBS/CaCl2 (0.002 M) pH 7.4 37 °C**				
	*k_KP_*	*N*	R^2^	*k_H_*	R^2^
Gel/Alg/MNP/EMF	0.053 ±0.029	0.758 ±0.111	0.995	0.876 ±0.132	0.994
Gel/Alg/MNP	0.515 ±0.118	0.173 ±0.058	0.993	1.181 ±0.019	0.882
Gel/Alg	0.603 ±0.012	0.125 ±0.001	0.998	1.305 ±0.004	0.849
	**CaCl2 (0.5 M) pH 5.5 25 °C**				
	*k_KP_*	*N*	R^2^	*k_H_*	R^2^
Gel/Alg/MNP/EMF	0.054 ±0.008	0.667 ±0.028	0.935	0.516 ±0.197	0.940
Gel/Alg/MNP	0.075 ±0.015	0.589 ±0.050	0.956	0.702 ±0.260	0.959
Gel/Alg	0.075 ±0.011	0.557 ±0.026	0.949	0.778 ±0.053	0.938

**Table 3:** Some examples of polymeric systems designed for CAF release and their corresponding highlights.

Polymer Matrix	CAF Incorporation	Kinetic Model	Highlights	Ref.
Alginate/starch Alginate/starch/xanthan Alginate/starch/xanthan/chitosan Alginate/whey protein	69.74–82.25%	Not applied	80% of CAF released after 30 min. The lowest level of release of CAF was obtained for alginate microcapsules with chitosan.	[28]
Alginate Alginate–Pectin Alginate–Carrageenan Alginate–Chitosan Alginate–Psyllium	68.94–83.68%	Not applied	More than 50% of the CAF was released in the first 5 min. Alginate systems with pectin or chitosan were the most favorable carrier systems.	[27]
Alginate Alginate/Chitosan	37.51–64.28%	Korsmeyer–Peppas	Fickian diffusion law was the prevalent mechanism. The total release of CAF was fast.	[32]
Colloidosome (alginate/CaCO_3_)	-	Higuchi; Korsmeyer–Peppas; Kopcha	CAF release reached a steady state after approximately 100 min	[31]
Beta-glucan, resistant starch, and beta-cyclodextrin microparticles	74.22–96.52%	Not applied	Caffeine release was more prevalent in simulated intestinal juice than gastric medium, displaying controlled release mechanism for such systems.	[78]
Tablets with linseed polysaccharides	-	Zero-order, first-order, Higuchi, Hixson–Crowell, Korsmeyer–Peppas	Buffers at pH 1.2, 6.8, 7.4, and DI water were used. Negligible drug release (<10%) at pH 1.2 (2 h). Higher and sustained release at pH 6.8 and 7.4 up to 16 h.	[79]
3D printed filaments of PVA/caffeine/paracetamol	4.7–9.5%	Not applied	100% of drug released in less than 360 min.	[82]
Chitosan querctin -poly(N-isopropylacrylamide) hydrogels	>85%	Second-order	After 30 min, at 40 °C and pH 2.0, 50% of the drug was released (burst effect). At pH 7 and 25 °C or 40 °C, sustained release was achieved.	[80]
Poly[N-isopropylacrylamide-co-(3-methacryloxypropyltrimethoxysilane)] (pNS)/silica nanoparticles (SiP)	-	Korsmeyer–Peppas	Release in 150 mL of deionized water (pH 6.8) or at pH 1.7. 93% release was achieved within 4 h under both pH conditions.	[73]
Electrospun nanofibers of PVA/caffeine at weight ratio 25:1	-	Korsmeyer–Peppas, Higuchi	Burst- caffeine release 100% within 60 s.	[76]
Cellulose membrane	8 mg/cm²	--	80% released in PBS after 5 min.	[77]
Magnetic beads of alginate, chlorpheniramine maleate, CoFe_2_O_4_ nanoparticles	up to 70%	--	Diffusion of drug from hydrogels was controlled by the medium’s pH; maximal release was 60% after ~3 h.	[81]
Cellulose acetate butyrate	7 wt%	Korsmeyer–Peppas	Sustained release over 48 h. 1.5 g/L of CAF released in PBS after 48 h.	[50]
Gel/Alg	15.4 mg/g_hydrogel_	Korsmeyer–Peppas	45 min, in PBS/0.002 M CaCl_2_, 37 °C: 8.3 ± 0.2 mg/g_hydrogel_ or 0.33 mM 45 min in PBS, 37 °C, without Ca^2+^ ions: 12.8 ± 0.2 mg/g_hydrogel_ or 0.72 mM	This work
Gel/Alg/MNP	18.5 mg/g_hydrogel_	Korsmeyer–Peppas	After 45 min in PBS/0.002 M CaCl_2_ at 37 °C under EMF, 6.1 ± 0.8 mg/g_hydrogel_ or 0.30 mM CAF was released. After 45 min in PBS at 37 °C without Ca^2+^ and with or without EMF, 13.3 ± 0.5 mg/g_hydrogel_ or 0.71 mM CAF was released.	This work

## Data Availability

The data presented in this study are available on request from the corresponding author.

## Supplementary Materials

The following supporting information can be downloaded at: https://www.mdpi.com/article/10.3390/polym15071757/s1, Figure S1: The discs of hydrogels were positioned inside the wells of a 24-well plate. 24 NdFeB magnets (0.4 T) were arranged in a 24-well plate with a north face oriented upwards. 24 NdFeB magnets (0.4 T) were arranged with the south face oriented downwards in another plate. The 24-well plate containing the hydrogels was positioned between the magnet plates; Figure S2: (a) UV-Vis absorption spectrum of CAF at 0.02 g L^−1^ and (b) CAF calibration curve performed at 272 nm; Figure S3: The experimental setup used for the diffusion assays of Ca^2+^ ions through the hydrogels from the lower compartment to the upper compartment; Figure S4: X-ray diffraction pattern of Fe_3_O_4_ nanoparticles synthesized by co-precipitation method; Figure S5: (a) and (b) AFM topographic images of the MNP deposited on Si wafers by spin coating and (c) size distribution of MNP determined by AFM images (n = 106); Figure S6: SEM images of hydrogels Gel/Alg in the (A) absence and (B) presence of caffeine; Figure S7: Transmission FTIR spectra of Gel/Alg and Gel/Alg/MNP films with pure gelatin (Gel), and pure alginate (Alg) in the region of (a) (b) 400- 4000 cm^−1^, and (c) (d) comparison of the FTIR spectra of the hydrogels in the presence or absence of caffeine in the region of 500-900 cm^−1^; Figure S8: Typical stress-strain curves determined for dried Gel/Alg, Gel/Alg/CAF, Gel/Alg/MNP and Gel/Alg/MNP/CAF hydrogels. The Young modulus (E) values were calculated as the slope of the initial region (dash lines); Figure S9: Thermogravimetric curves and the corresponding 1st derivatives determined for (a) Gel/Alg, (b) Gel/Alg/CAF, (c) Gel/Alg/MNP and (d) Gel/Alg/MNP/CAF; Figure S10: (a) OCP measurements recorded in 0.1 mol L^−1^ KCl solution using the ISME-Ca^2+^ sensor in the 0.1 µmol L^−1^ to 0.5 mol L^−1^ CaCl_2_ concentration range before (black line) and after (red line) Ca^2+^ ions diffusion experiments using the 2-compartment cell. Inset: Calibration plots (E vs. pCa^2+^). (b) OCP measurements recorded using the ISME-Ca^2+^ sensor in 0.1 mol L^−1^ KCl solution during the addition of 1 mmol L^−1^ CAF (red line) and 1 mmol L^−1^ Ca^2+^ + 1 mmol L^−1^ CAF (blue line) solutions, as indicated by the arrows; Figure S11: Cumulative release of caffeine from the hydrogels in the absence (green and red symbols) and presence (blue) of MNP and EMF at (a) pH 5.5 and 25 °C, (b) pH 5.5 and 37 °C, (c) PBS buffer pH 7.4 and 37 °C, (d) PBS/CaCl 2 (0.002 mol L^−1^ ) and 37 °C, and (e) CaCl 2 0.5 mol L^−1^ and 25 °C. Solid lines are guides for the eyes. Solid lines were obtained by curve fitting; Figure S12: Cumulative CAF release as a function of square root of time (t^0.5^) from hydrogels in the absence and presence of EMF at (a) pH 5.5 and 25 °C, (b) pH 5.5 and 37 °C, (c) PBS buffer pH 7.4 and 37 °C, (d) PBS/CaCl_2_ (0.002 mol L^−1^) and 37 °C, and (e) CaCl_2_ 0.5 mol L^−1^ and 25 °C. The solid lines correspond to the fittings to the Higuchi model; Figure S13: Statistical analysis for kKP and n values obtained from the Korsmeyer-Peppas fitting curves (Figure S11). All data were analyzed by ANOVA (One-way analysis of variance) to assess differences among Gel/Alg, Gel/Alg/MNP and Gel/Alg/MNP/EMF. Results are expressed mean ± SD and their *p* values (* *p* < 0.05, ** *p* < 0.005 and *** *p* < 0.0005); Figure S14: Swelling degree values for Gel/Alg and Gel/Alg/MNP obtained in Milli-Q water (pH 5.5) and 25 °C, CaCl_2_ (0.5 mol L^-1^) and 25 °C, Milli-Q water and 37 °C, PBS (pH 7.4) and 37 °C; Figure S15: Optical microscopy images of SH-SY5Y cells treated with CAF in the concentration range from 0.2 mM to 100 mM for 24 h. The scale bar corresponds to 400 µm.
