# Supplementary material for: Caffeine Release from Magneto-Responsive Hydrogels Controlled by External Magnetic Field and Calcium Ions and Its Effect on the Viability of Neuronal Cells
Source: Polymers (Basel). 2023 Mar 31;15(7):1757. doi: 10.3390/polym15071757 (PMC10097041; doi:10.3390/polym15071757)

Supplementary Material

Caffeine release from magneto-responsive hydrogels controlled by external magnetic field and Calcium ions and its effect on the viability of neuronal cells

Emilli C. G. Frachini[1], Jéssica S. G. Selva[1], Paula C. Falcoswki[1], Jean B. Silva[2], Daniel R. Cornejo[3], Mauro Bertotti[1], Henning Ulrich[2,*] and Denise F. S. Petri[1,*]

Figure S1 - The discs of hydrogels were positioned inside the wells of a 24-well plate. 24 NdFeB magnets (0.4 T) were arranged in a 24-well plate with a north face oriented upwards. 24 NdFeB magnets (0.4 T) were arranged with the south face oriented downwards in another plate. The 24-well plate containing the hydrogels was positioned between the magnet plates.

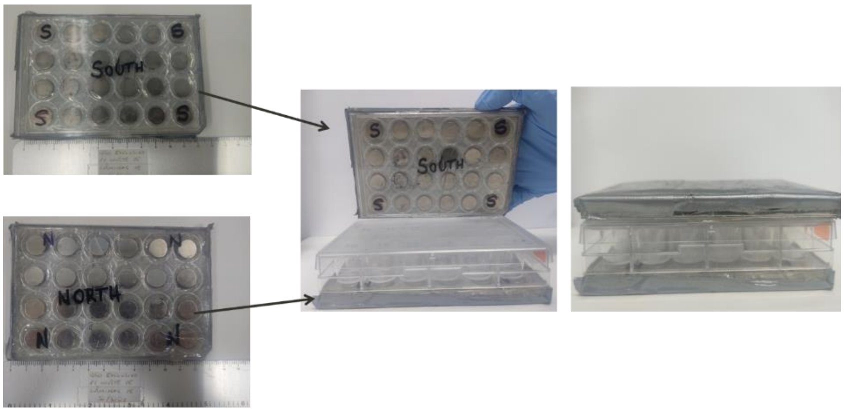

Figure S2 - (a) UV-Vis absorption spectrum of CAF at 0.02 g L^{-1} and (b) CAF calibration curve performed at 272 nm.

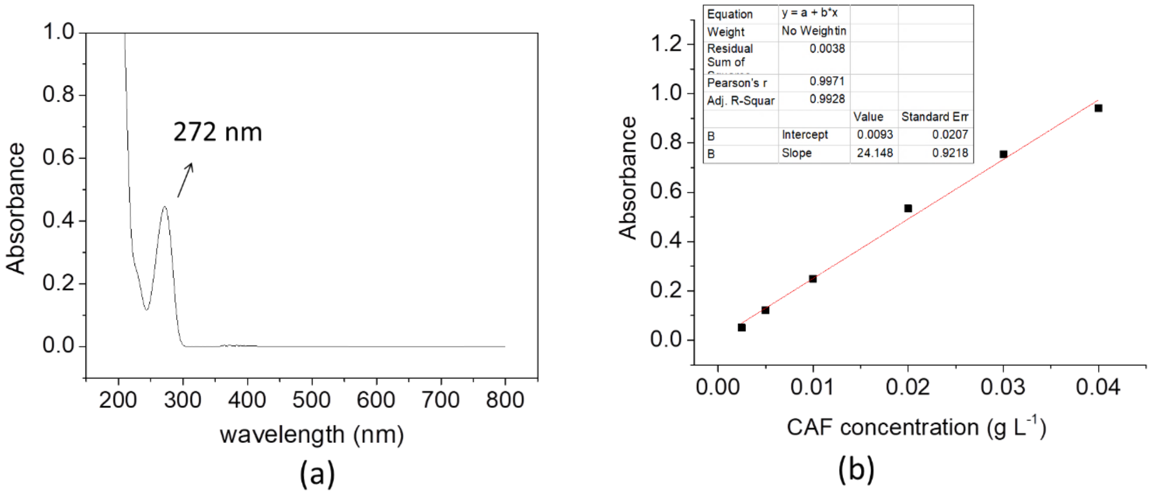

Figure S3 - The experimental setup used for the diffusion assays of Ca^{2+} ions through the hydrogels from the lower compartment to the upper compartment.

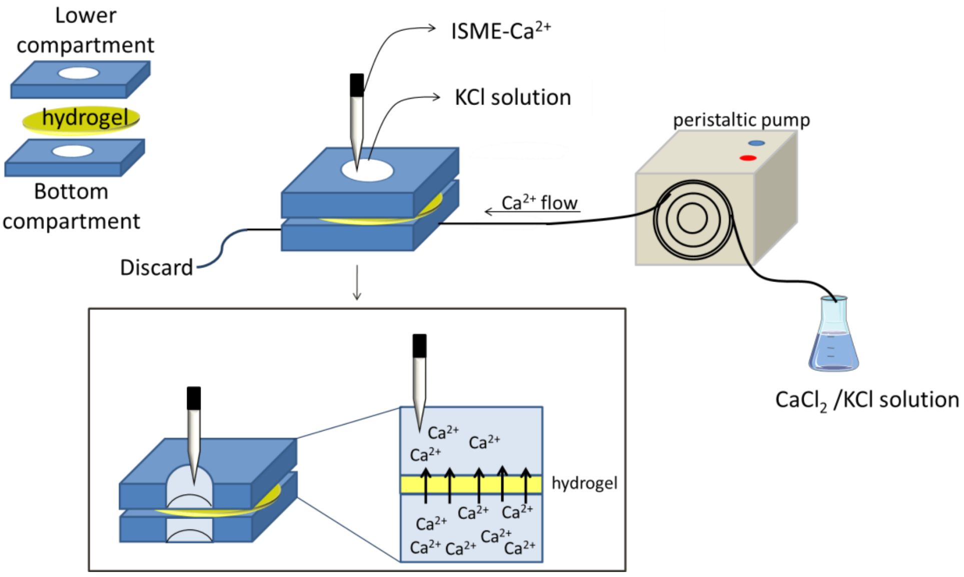

Figure S4 - X-ray diffraction pattern of Fe_3O_4 nanoparticles synthesized by co-precipitation method.

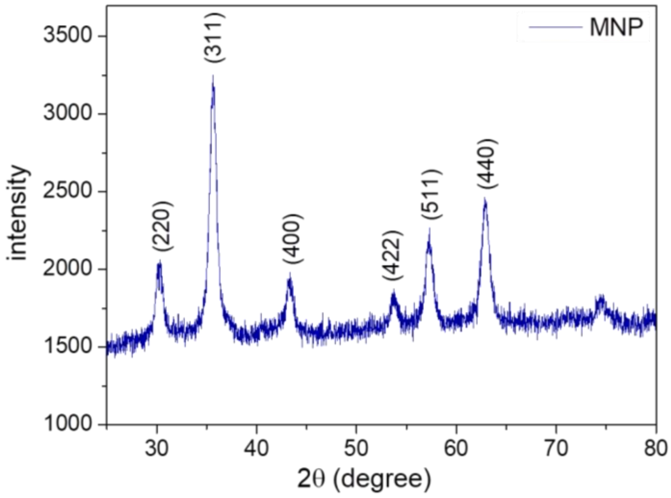

Figure S5 - (a) and (b) AFM topographic images of the MNP deposited on Si wafers by spin coating and (c) size distribution of MNP determined by AFM images (n = 106).

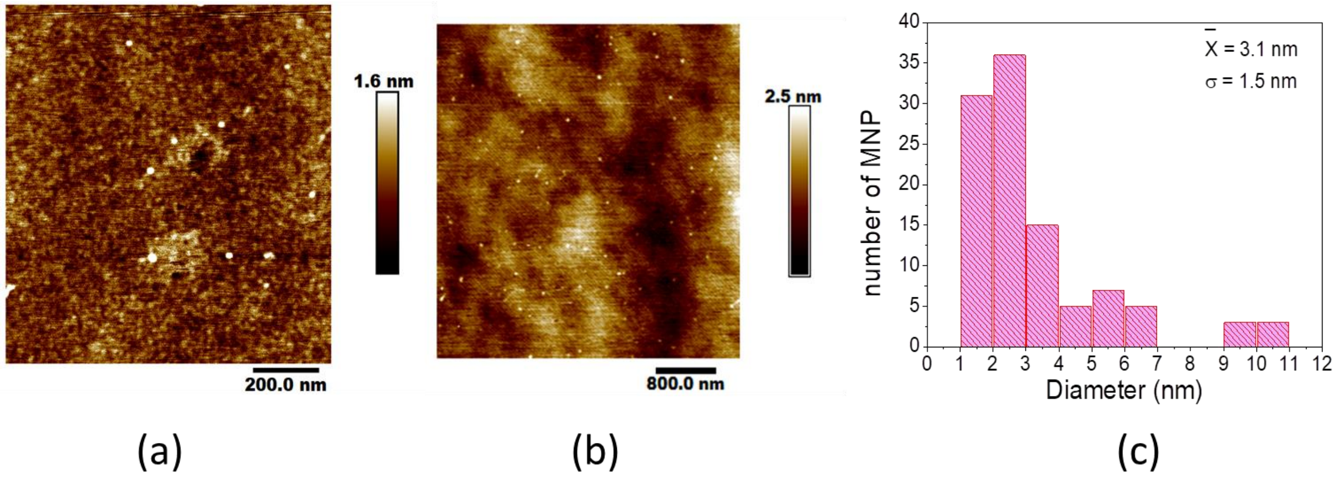

Figure S6 - SEM images of hydrogels Gel/Alg in the (A) absence and (B) presence of caffeine.

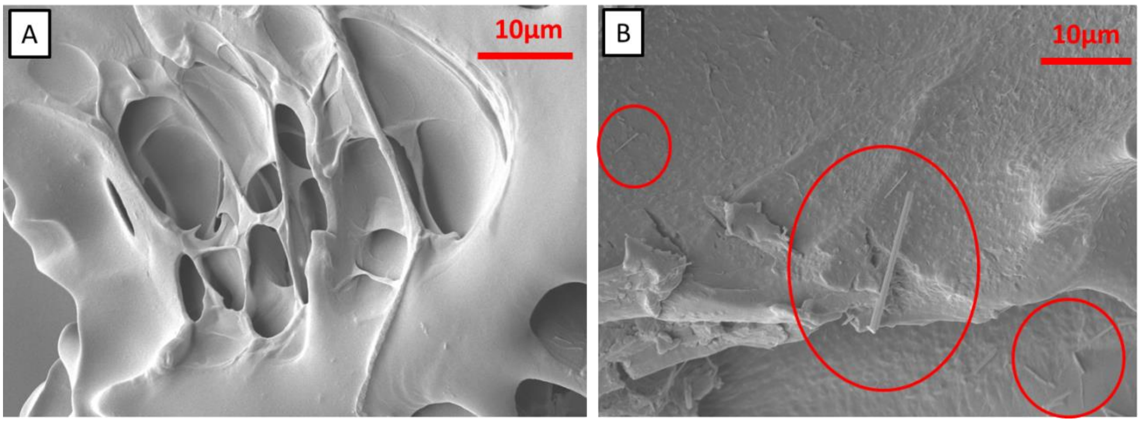

Figure S7 - Transmission FTIR spectra of Gel/Alg and Gel/Alg/MNP films with pure gelatin (Gel), and pure alginate (Alg) in the region of (a) (b) 400- 4000 cm-1, and (c) (d) comparison of the FTIR spectra of the hydrogels in the presence or absence of caffeine in the region of 500-900 cm-1.

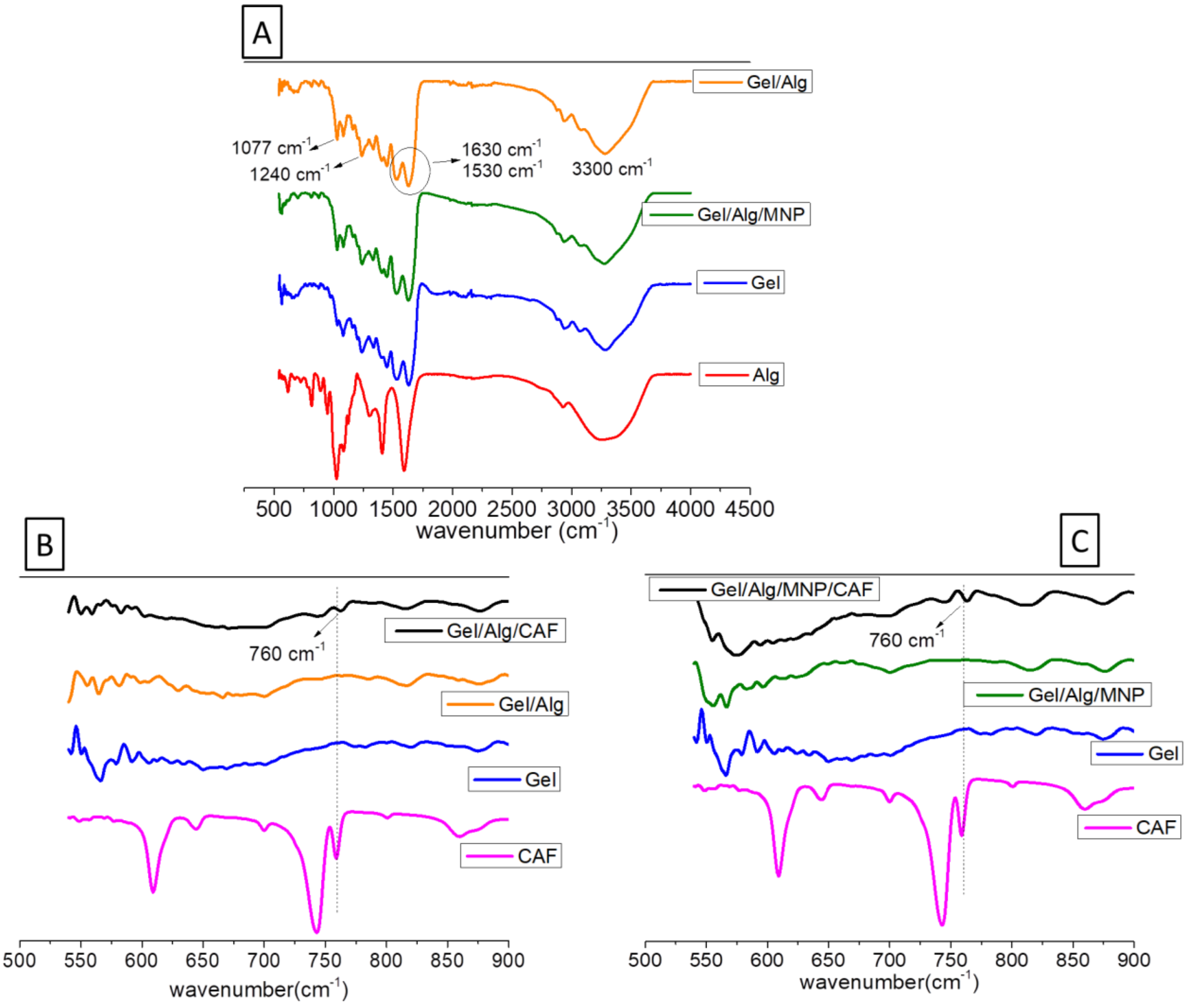

Figure S8 - Typical stress-strain curves determined for dried Gel/Alg, Gel/Alg/CAF, Gel/Alg/MNP and Gel/Alg/MNP/CAF hydrogels. The Young modulus (E) values were calculated as the slope of the initial region (dash lines).

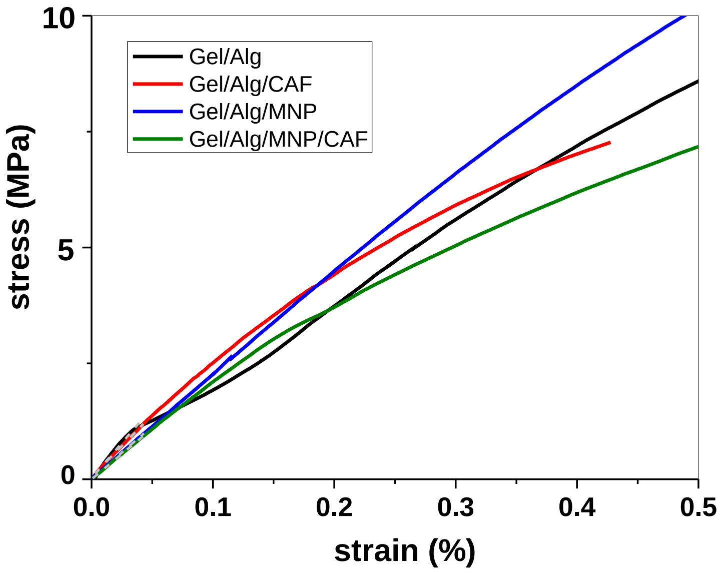

Figure S9 - Thermogravimetric curves and the corresponding 1st derivatives determined for (a) Gel/Alg, (b) Gel/Alg/CAF, (c) Gel/Alg/MNP and (d) Gel/Alg/MNP/CAF.

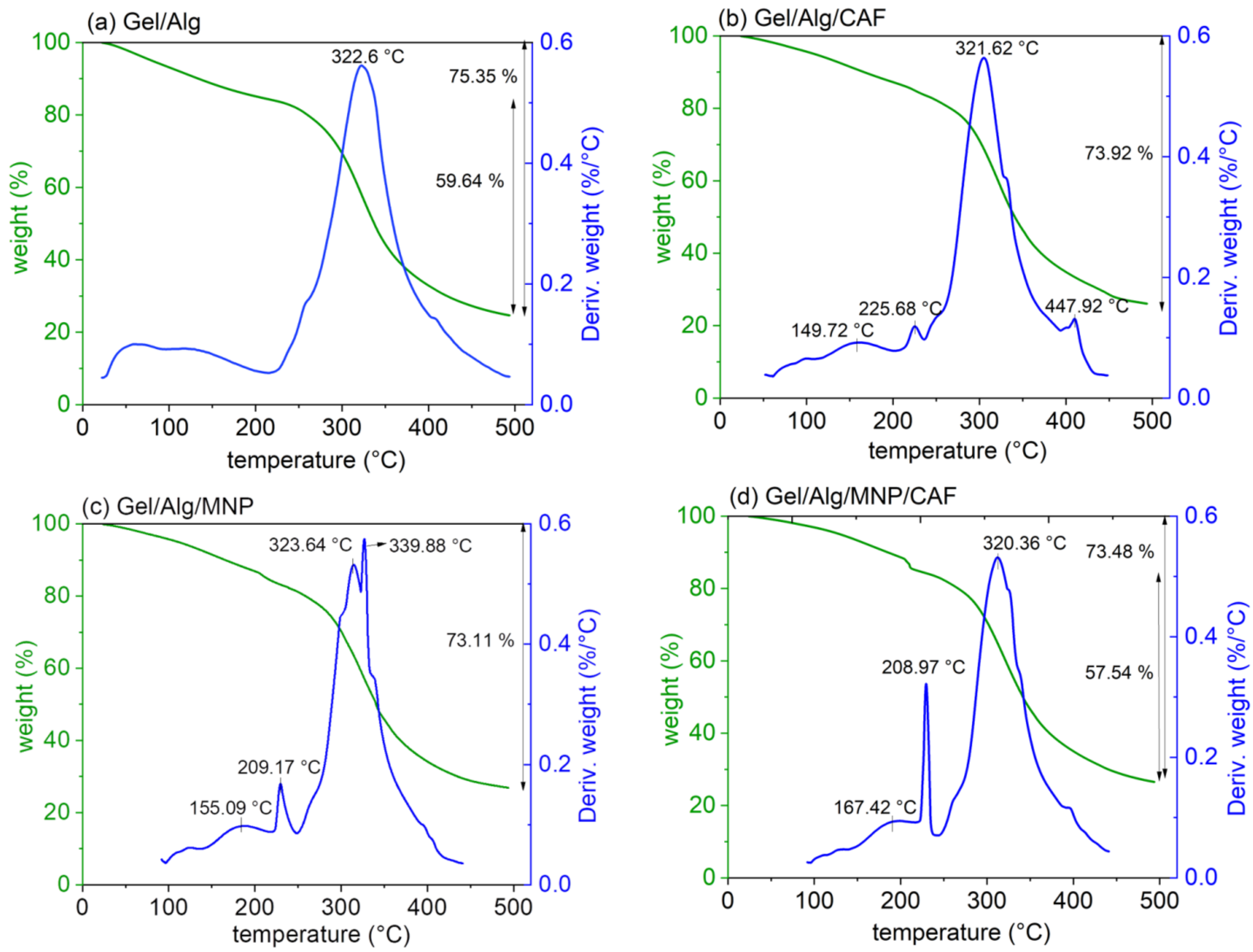

Figure S10 - (a) OCP measurements recorded in 0.1 mol L^{-1} KCl solution using the ISME-Ca^{2+} sensor in the 0.1 μmol L^{-1} to 0.5 mol L^{-1} $CaCl_2$ concentration range before (black line) and after (red line) Ca^{2+} ions diffusion experiments using the 2-compartment cell. Inset: Calibration plots (E vs pCa^{2+}). (b) OCP measurements recorded using the ISME-Ca^{2+} sensor in 0.1 mol L^{-1} KCl solution during the addition of 1 mmol L^{-1} CAF (red line) and 1 mmol L^{-1} Ca^{2+} + 1 mmol L^{-1} CAF (blue line) solutions, as indicated by the arrows.

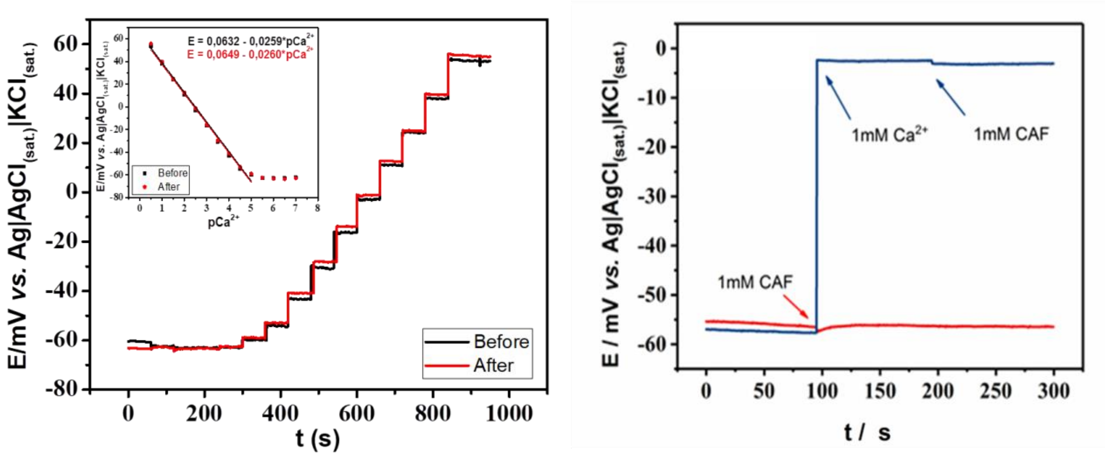

(a) (b)

Figure S11 - Cumulative release of caffeine from the hydrogels in the absence (green and red symbols) and presence (blue) of MNP and EMF at (a) pH 5.5 and 25°C, (b) pH 5.5 and 37°C, (c) PBS buffer pH 7.4 and 37°C, (d) PBS/CaCl 2 (0.002 mol L -1) and 37°C, and (e) CaCl 2 0.5 mol L -1 and 25°C. Solid lines are guides for the eyes. Solid lines were obtained by curve fitting.

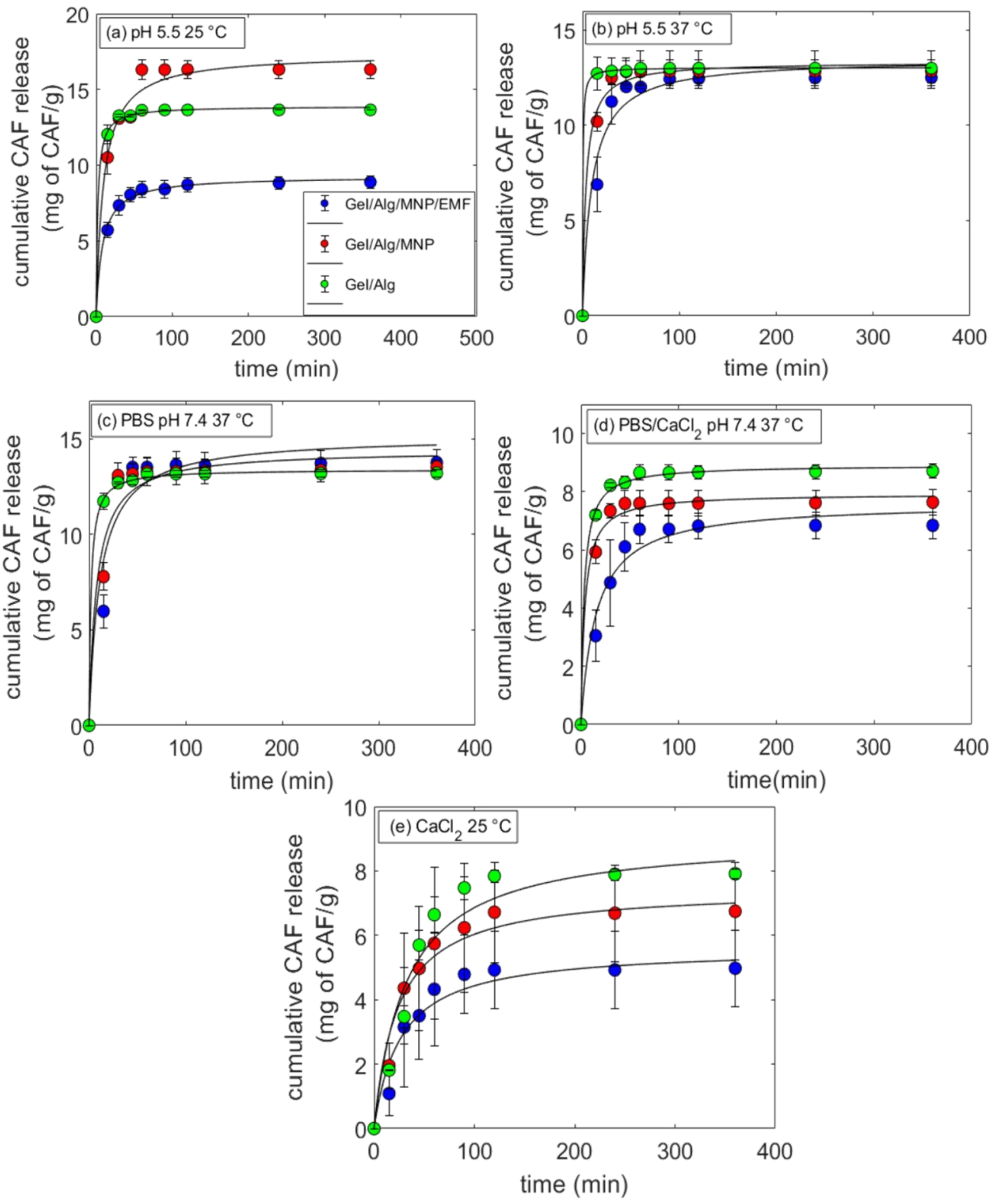

Figure S12 - Cumulative CAF release as a function of square root of time ($t^{0.5}$) from hydrogels in the absence and presence of EMF at (a) pH 5.5 and 25 °C, (b) pH 5.5 and 37 °C, (c) PBS buffer pH 7.4 and 37 °C, (d) PBS/$CaCl_2$ (0.002 mol L^{-1}) and 37 °C, and (e) $CaCl_2$ 0.5 mol L^{-1} and 25 °C. The solid lines correspond to the fittings to the Higuchi model.

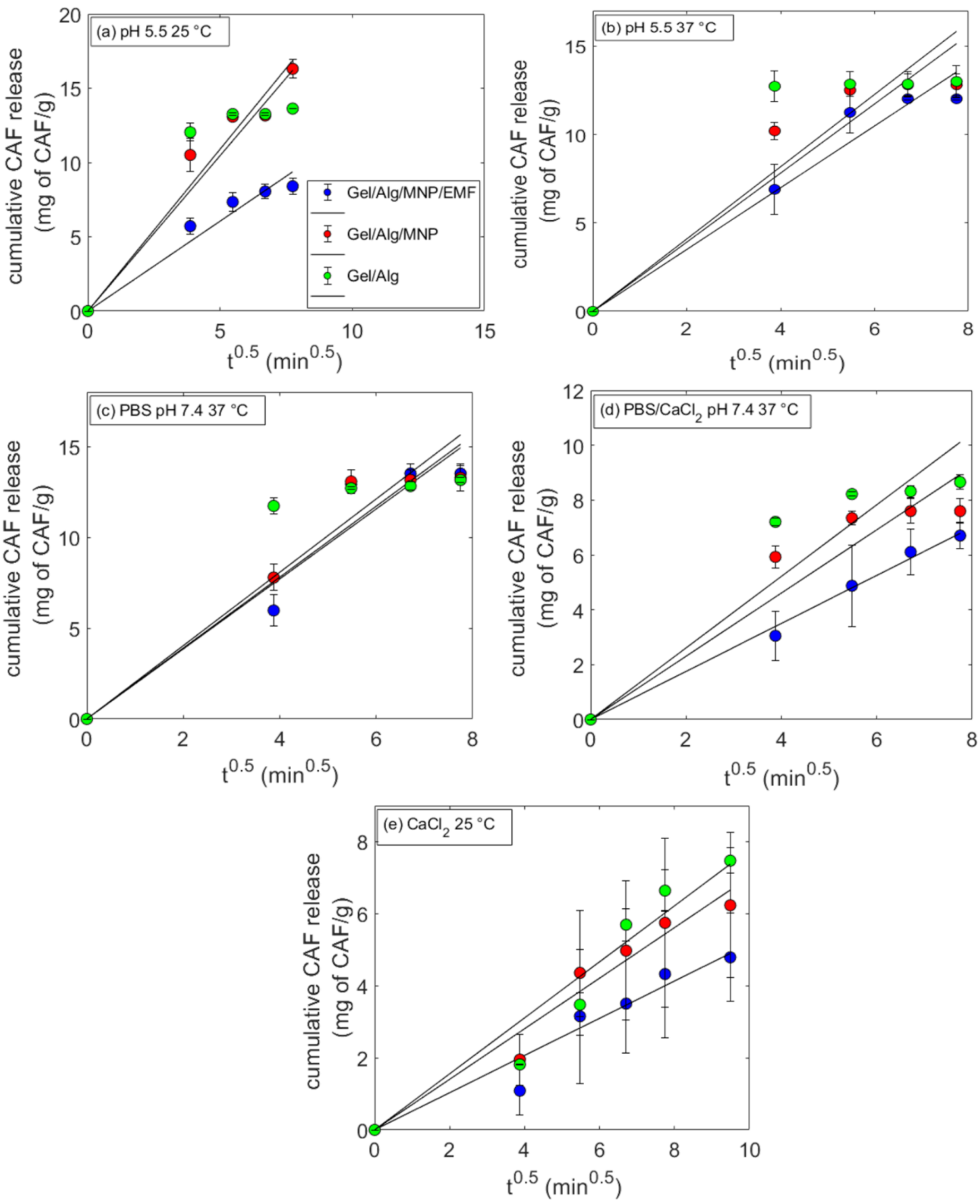

Figure S13 - Statistical analysis for k_{KP} and n values obtained from the Korsmeyer-Peppas fitting curves (**Figure S11**). All data were analyzed by ANOVA (One-way analysis of variance) to assess differences among Gel/Alg, Gel/Alg/MNP and Gel/Alg/MNP/EMF. Results are expressed mean ± SD and their p values (*p < 0.05, **p < 0.005 and ***p < 0.0005).

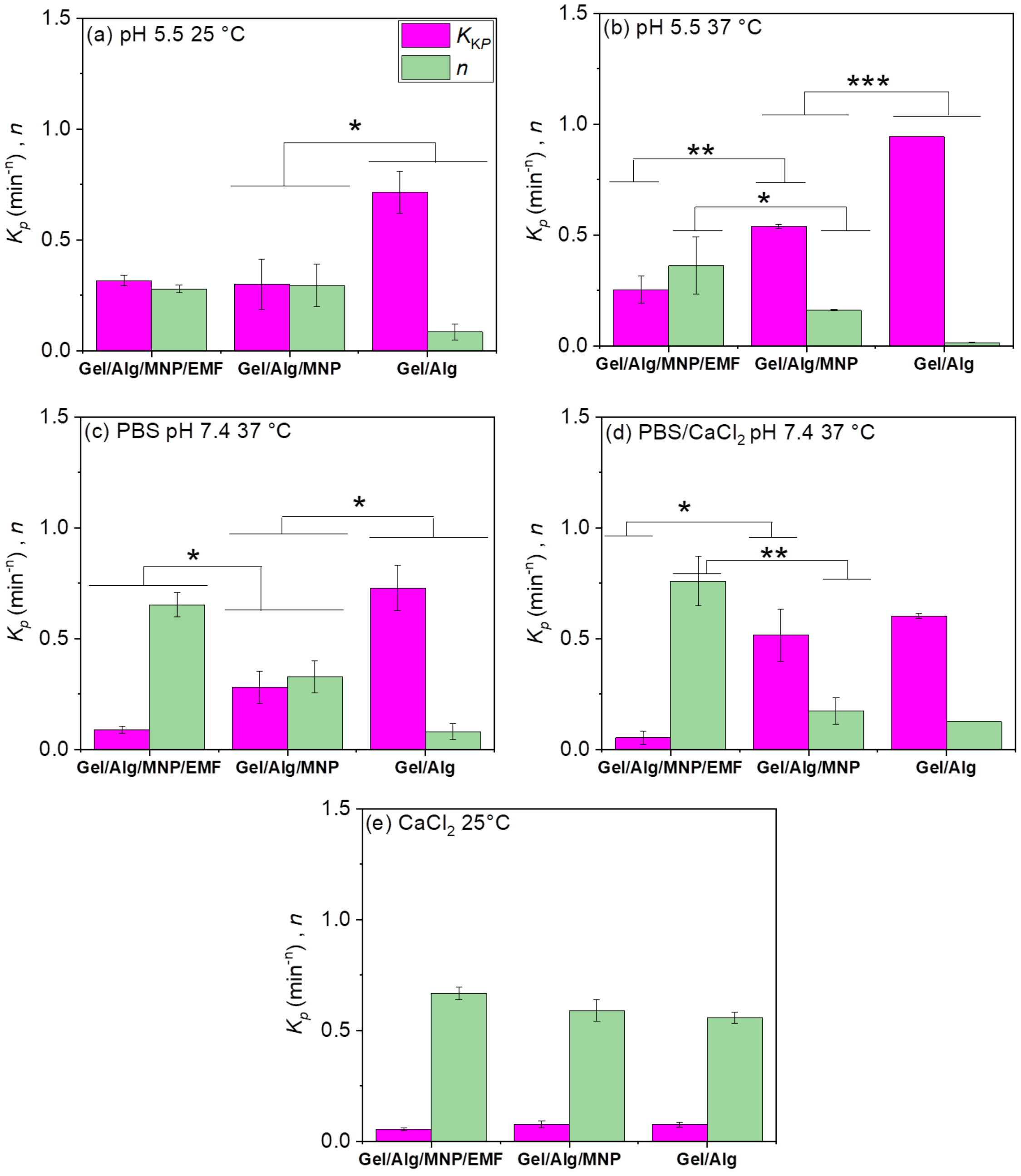

Figure S14 – Swelling degree values for Gel/Alg and Gel/Alg/MNP obtained in Milli-Q water (pH 5.5) and 25 °C, $CaCl_2$ (0.5 mol L^{-1}) and 25 °C, Milli-Q water and 37 °C, PBS (pH 7.4) and 37 °C.

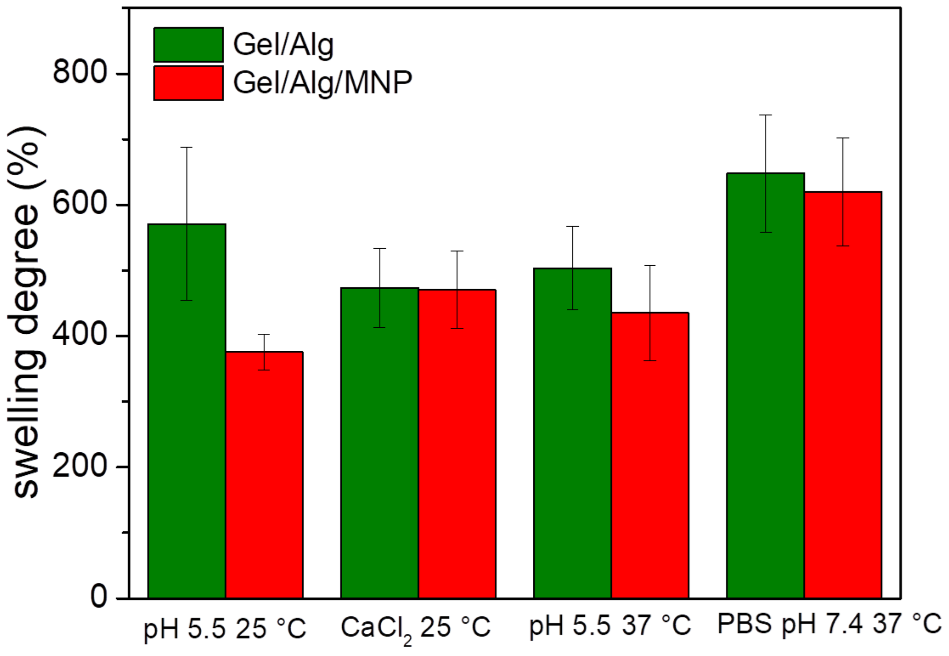

Figure S15 – Optical microscopy images of SH-SY5Y cells treated with CAF in the concentration range from 0.2 mM to 100 mM for 24 h. The scale bar corresponds to 400 μm.

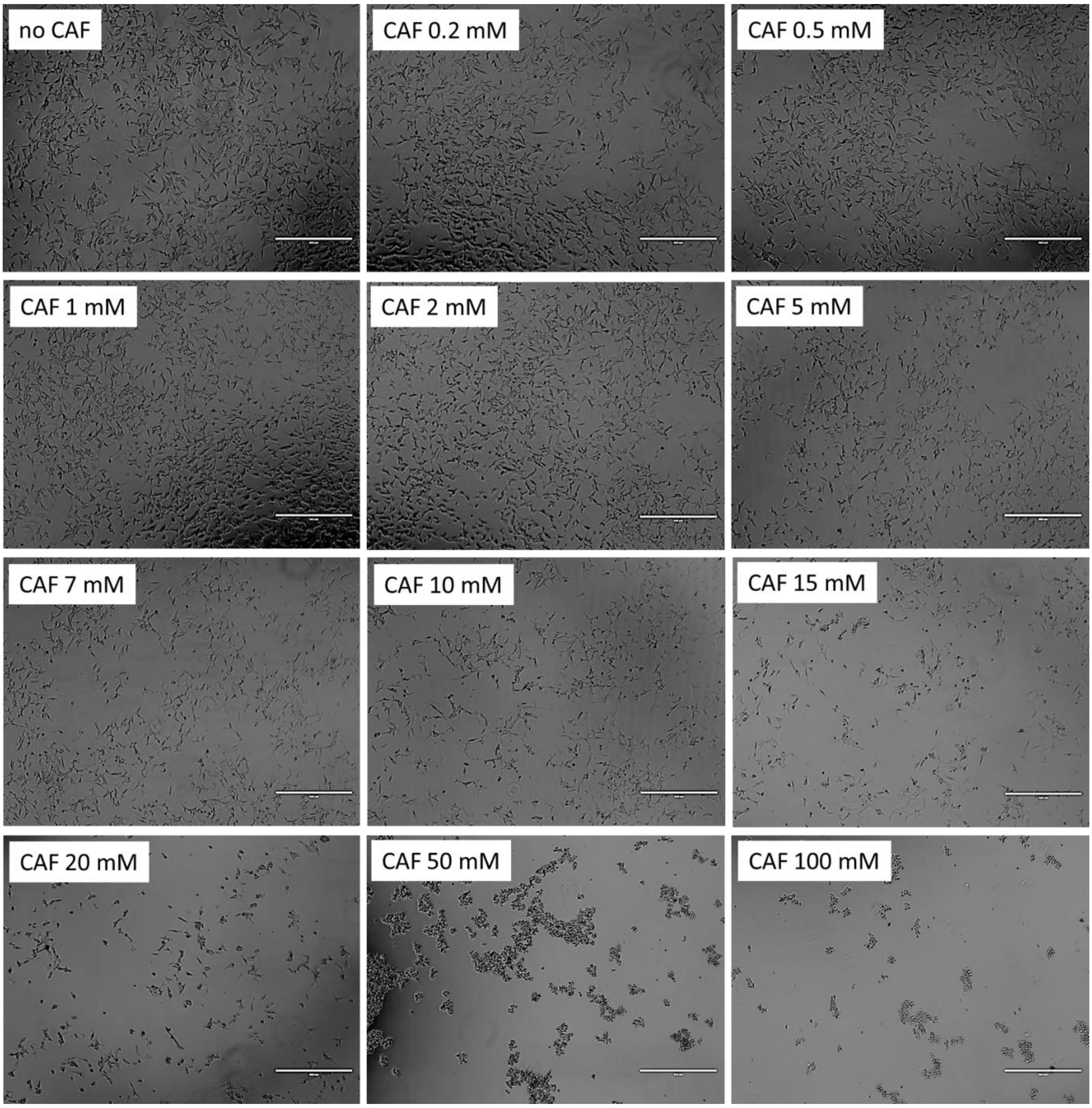

Supplement: Supplementary file 1 [file polymers-15-01757-s001.zip › polymers-2289575-supplementary.pdf]
